# EEG-based clinical decision support system for Alzheimer's disorders diagnosis using EMD and deep learning techniques

**DOI:** 10.3389/fnhum.2023.1190203

**Published:** 2023-08-31

**Authors:** Khalil AlSharabi, Yasser Bin Salamah, Majid Aljalal, Akram M. Abdurraqeeb, Fahd A. Alturki

**Affiliations:** Electrical Engineering Department, College of Engineering, King Saud University, Riyadh, Saudi Arabia

**Keywords:** Alzheimer's disease, artificial neural network, convolutional neural network, deep learning, empirical mode decomposition, K-fold, leave-one-subject-out cross-validation

## Abstract

**Introduction:**

Despite the existence of numerous clinical techniques for identifying neurological brain disorders in their early stages, Electroencephalogram (EEG) data shows great promise as a means of detecting Alzheimer's disease (AD) at an early stage. The main goal of this research is to create a reliable and accurate clinical decision support system leveraging EEG signal processing to detect AD in its initial phases.

**Methods:**

The research utilized a dataset consisting of 35 neurotypical individuals, 31 patients with mild AD, and 22 patients with moderate AD. Data were collected while participants were at rest. To extract features from the EEG signals, a band-pass filter was applied to the dataset and the Empirical Mode Decomposition (EMD) technique was employed to decompose the filtered signals. The EMD technique was then leveraged to generate feature vectors by combining multiple signal features, thereby enhancing diagnostic performance. Various artificial intelligence approaches were also explored and compared to identify features of the extracted EEG signals distinguishing mild AD, moderate AD, and neurotypical cases. The performance of the classifiers was evaluated using k-fold cross-validation and leave-one-subject-out (LOSO) cross-validation methods.

**Results:**

The results of this study provided valuable insights into potential avenues for the early diagnosis of AD. The performance of the various offered methodologies has been compared and evaluated by computing the overall diagnosis precision, recall, and accuracy. The proposed methodologies achieved a maximum classification accuracy of 99.9 and 94.8% for k-fold and LOSO cross-validation techniques, respectively.

**Conclusion:**

The study aims to assess and compare different proposed methodologies and determine the most effective combination approach for the early detection of AD. Our research findings strongly suggest that the proposed diagnostic support technique is a highly promising supplementary tool for discovering prospective diagnostic biomarkers that can greatly aid in the early clinical diagnosis of AD.

## 1. Introduction

The term “neurological brain disorders” refers to any conditions that affect the brain or another area of the nervous system, and AD is one of the most prevalent neurological brain disorders worldwide. AD is a type of neurodegenerative disease characterized by a progressive loss of neurological, mental, and cognitive functions, including memory, changes in judgment, behavior, and emotions (Fonteijn et al., 2012; Ghanemi, 2015; Caruso et al., 2018). It also represents the primary cause of dementia because it damages brain neurons, especially the axons, by destroying neurotransmitters crucial for memory storage and message transmission to the brain (Miltiadous et al., 2021). Neurological illnesses affect hundreds of millions of individuals globally, and they pose a threat to global public health. In 2005, World Health Organization (WHO) estimated that 0.379% of the global population was predicted to have dementia, and by 2030, that number was projected to rise to 0.556% (WHO, 2006). According to their most recent data (fact sheet December, 2017), there are 47.5 million instances of dementia, with AD possibly being a factor in 60–70% of those cases. According to Lipton et al. (2016), there were 29.8 million people worldwide with Alzheimer's disease in 2015. From a prognostic and therapeutic standpoint, diagnosing the various stages of AD is crucial, and clinically speaking, it is crucial to distinguish them (Shimizu et al., 2005). However, the diagnosis of neurological brain disorders is still primarily done manually by neurologists or other medical specialists, who are scarcely available. Neurologists may need many hours in some circumstances to make a final diagnosis for a single patient.

Aa a result, there is an urgent need for a biomarker that is reliable, ubiquitous, specific, and cost-effective for diagnosing AD types and tracking disease progression and treatment response because early identification is crucial for commencing treatment that can reduce disease progression. In recent years, researchers in the multidisciplinary fields of bioengineering and neurology have made significant attempts to construct a clinical decision-support system capable of swiftly and accurately diagnosing the early stages of AD. For this, the researchers employ all of the information offered by EEG signals since they are simple, relatively inexpensive, and generally available. Several EEG signal characteristics enable EEG to have greater potential to find AD early. For example, studies have shown that individuals with AD exhibit abnormalities in their EEG signal power and coherence, particularly in the alpha and theta frequency bands. These abnormalities can be detected even in the early stages of the disease, making EEG a promising tool for early detection (Jeong, 2004; Babiloni et al., 2016). Individuals with AD often exhibit changes in their EEG spectral density, particularly in the so-called delta, theta, and alpha frequency bands. These changes have been observed in the early stages of the disease before a significant cognitive decline occurs (Jeong, 2004; Babiloni et al., 2016). Other studies have identified specific EEG biomarkers that are associated with AD, such as reduced alpha power, increased theta power, and reduced coherence between brain regions. These biomarkers can be used to differentiate individuals with AD from healthy controls and may have the potential to predict the onset of AD in asymptomatic individuals (Poil et al., 2013). Moreover, EEG has a high temporal resolution, which means that it can detect changes in brain activity with millisecond precision. This high temporal resolution makes it possible to detect subtle changes in brain function that may be indicative of early AD (Babiloni et al., 2016). Overall, these EEG characteristics make it a promising tool for the early detection of AD and monitoring disease progression, and EEG data processing is thought to be effective for discriminating between distinct types of AD and for making early diagnoses of those types (AlSharabi et al., 2022).

Accordingly, the study focused on how to employ EEG signal processing to identify and distinguishing between mild AD, moderate AD, and neurotypical signals to aid medical practitioners in early AD diagnosis. Recently, various signal processing and artificial intelligence techniques have been developed for EEG feature extraction and classification. These techniques have been explored to create a Computer-Aided Diagnosis (CAD) system. The objective of this system is to automate the analysis of brain signals and support neurologists in diagnosing various neurological disorders, including were presented and discussed for the development of a Computer-Aided Diagnosis (CAD) system capable of automatically analyzing brain signals and assisting neurologists in diagnosing neurological disorders such as Autism spectrum disorder (ASD) (Djemal et al., 2017), epilepsy disorders (ED) (AlSharabi et al., 2016), both Autism spectrum disorder and epilepsy disorders (Alturki et al., 2019, 2020, 2021), and Parkinson disease (Aljalal et al., 2022a,b). As a result, numerous academics have been working on clinical decision support systems to diagnose the phases of AD by studying brain signals from patients. Morabito et al. (2016) proposed using Convolutional Neural Networks (CNN) in deep learning to build acceptable sets of EEG signal features. The proposed system employed a series of convolutional subsampling layers to generate a multivariate assembly of unique patterns for classifying sets of EEG from different subject classes. The system achieved an accuracy of 85%, which was not considered encouraging. On the other hand, Cassani et al. (2017) developed an automated EEG-based (AD) diagnostic system based on an automated artifact removal (AAR) algorithm and a low-density EEG setup (seven channels). The system computed common EEG parameters, including spectral power and coherence, as well as amplitude-modulation properties, and used Support Vector Machine (SVM) for classification. The maximum accuracy achieved by the system was 91.4%. However, the study's fundamental limitation was the low-density EEG setup, which did not record all brain activities.

Ieracitano et al. (2020) developed a multi-modal machine learning-based approach for automatically classifying EEG recordings in dementia. The approach used features extracted using the Continuous Wavelet Transform (CWT) and bispectrum (BiS) features, as well as Multi-Layer Perceptron (MLP) and SVM classifiers. The proposed approach achieved an accuracy of 96.95%. Trambaiolli et al. (2017) achieved an accuracy of 91.18% in EEG spectrum measurements and classification using eight distinct feature selection algorithms and an SVM classifier. Simons et al. (2015) investigated the quadratic sample entropy (QSE) to detect changes in EEG signals and classified them using linear discriminant analysis (LDA). However, the proposed approach had a diagnostic accuracy of only 77.27%, which was considered the main disadvantage of the study. Bevilacqua et al. (2015) tested various classifiers for discriminating between normal and AD patients using EEG data. They used three approaches to reduce feature dimensionality, including Support Vector Machines Recursive Features Elimination (SVMRFE), Principal Component Analysis (PCA), and a correlation-based method. The study compared two distinct SVM setups, three Error-Back Propagation Multi-Layer Perceptron Artificial Neural Network (ANN) configurations, and five classifiers MLP-ANN. Despite using three feature extraction strategies and five classifiers, the accuracy was not considered promising, but their method achieved a diagnostic accuracy of 86%. Fiscon et al. (2018a,b) developed a diagnostic system for AD that used Fourier and wavelet analysis for feature extraction and a tree-based classifier (J48). The proposed approach achieved a maximum diagnostic accuracy of 80.2%.

Simons et al. (2018) used Fuzzy Entropy (FuzzyEn) to analyze EEG AD data to reach a maximum diagnostic accuracy of 86.36%. The biggest problem with this study is the minimal number of individuals used. Ruiz-Gómez et al. (2018a) processed EEG signals using the relative and median frequency, individual alpha frequency, spectral entropy, Lempel-Ziv complexity, central tendency measure, sample entropy, fuzzy entropy, and automutual information to diagnose AD. The study used LDA, quadratic discriminant analysis (QDA), and MLP-ANN classifiers and achieved a maximum diagnostic accuracy of 78.43%. However, the classification accuracy was not considered promising. Another study (Ruiz-Gómez et al., 2018b) estimated the Cross-Approximate Entropy (Cross-ApEn) and Cross-Sample Entropy (Cross-SampEn) of EEG data and used QDA, SVM, and Decision Tree (DT) as classifiers. The proposed method achieved a maximum diagnostic accuracy of 82.35%. Triggiani et al. (2017) used exact low-resolution brain electromagnetic tomography (eLORETA) to quantify the power and functional connectivity of cortical sources in various brain areas and an ANN classifier to achieve 76.7% diagnostic accuracy. The study analyzed the brain areas individually, which was a benefit, but the precision was not considered encouraging. Houmani et al. (2018) used epoch-based entropy and bump modeling to extract features from EEG signals and classify them using an SVM classifier. The proposed diagnostic method achieved an accuracy of 91.6%. Maturana-Candelas et al. (2019) estimated the multiscale sample entropy (MSE) and refined the multiscale spectral entropy (rMSSE) from EEG signals to demonstrate their irregularity and complexity. They used a QDA classifier and achieved a maximum diagnostic accuracy of 79.1%. However, the study's main issue was its significant gender imbalance, with 177 females and only 76 males included.

Amezquita-Sanchez et al. (2019) proposed an EEG-based CAD system for AD using integrated multiple signal classification and empirical wavelet transforms, nonlinear features from chaos theory such as fractality dimension (FD), and the enhanced probabilistic neural network (EPNN) model for classification. The proposed approach achieved a maximum diagnosis accuracy of 90.3%. Rodrigues et al. (2016) developed a classification methodology for EEG signals to improve discrimination amongst patients at varying stages of the illness, Mild Cognitive Impairment (MCI) patients, and non-patients. They used the Discrete Wavelet Transform (DWT) for feature extraction and surrogate DT for classification. Their proposed system achieved a maximum accuracy of 95.45 and 94.88% for k-fold and LOSO cross-validation, respectively. Cura et al. (2021) utilized EMD, Ensemble EMD, and DWT for feature extraction and employed DT, SVM, k-Nearest Neighbor (kNN), and Random Forests (RF) for classification. Their proposed system achieved a maximum accuracy of 96.5%. Miltiadous et al. (2021) developed a classification system for diagnosing AD and Frontotemporal Dementia using EEG signals. They extracted features using the Fast Fourier Transform (FFT) and classified them using six machine-learning approaches. The suggested approach achieved a classification accuracy of 86.3%. However, the study used a small clinical sample of 18 or 20 patients for each classification task. Recently, Safi and Safi (2021) utilized Hjorth parameters, along with other common features, to enhance the detection accuracy of AD in the early stages from EEG signals. They employed DWT and EMD for feature extraction and SVM, KNN, and LDA for classification. Their proposed system achieved a maximum accuracy of 97.64 and 81.08% for k-fold and LOSO cross-validation, respectively. Pirrone et al. (2022) developed a simple and effective method to extract features using a Finite Response Filter (FRF) and PCA to distinguish between patients with AD, mild cognitive impairment (MCI), and healthy controls (HC). They calculated the power intensity for high and low-frequency bands and used RNN for classification. Their proposed system achieved a maximum accuracy of 97%. Alessandrini et al. (2022) developed an automatic classification method that can effectively handle EEG data affected by artifacts. The study employed the robust principal component analysis (RPCA) algorithm for filtering and PCA for feature extraction, while the RNN technique was utilized for classification. Their proposed system achieved a maximum accuracy of 97.9%.

Finally, Tsai et al. (2012) researched the relationship between changes in the complexity of EEG signals and dementia. They demonstrated that quantitative EEG analysis, which involved a combination of the EMD detrending procedure and sample entropy, can reveal a correlation between changes in the complexity of EEG signals and the severity of dementia. This may offer a non-invasive, low-cost, and objective tool for the clinical evaluation and follow-up of dementia patients. As a result, the method's universality cannot be established. It is important to note that developing an optimal clinical decision support system for the early diagnosis of AD is crucial for the later therapeutic and healthcare stages. However, AD diagnosis is still primarily performed manually by neurologists or medical experts, who are limited in number, and it is a time-consuming process. Most of the literature reviewed has employed traditional approaches with low accuracy in diagnosis. To date, no study has yet utilized EEG signal decomposition, feature extraction, and classification to construct a reliable and effective clinical decision support system for identifying the stages of AD. Therefore, this study aims to address these limitations by combining and integrating optimal techniques for EEG signal decomposition, feature extraction, cross-validation, and classification. The key goal is to improve the diagnosis and detection of various stages of AD (mild and moderate) from neurotypical signals, or to distinguish between two or three classes of AD stages.

The present study aims to develop a clinical decision support system for neurologists to diagnose AD automatically, quickly, and reliably using EEG data processing. The proposed approach was validated using datasets from mild and moderate AD patients, as well as neurotypical individuals. The pre-processing stage involved filtering the EEG datasets using a band-pass elliptic filter. The filtered signal was then decomposed into intrinsic mode functions and residual using the EMD method. Several signal features, such as approximate entropy, signal energy, logarithmic band power, mean frequency, Norm, peak-to-peak value, and zero-crossing rate, were extracted from the EMD output to construct a feature matrix and improve diagnostic accuracy. Five classification issues were addressed using the extracted features from three datasets: neurotypical individuals vs. mild AD, neurotypical individuals vs. moderate AD, mild AD vs. moderate AD, neurotypical individuals vs. mild and moderate AD, and neurotypical individuals vs. mild AD vs. moderate AD. Two types of artificial intelligence approaches were used: machine learning (LDA, SVM, and RF) and deep learning (ANN, RNN, and CNN). The performance of the proposed approaches was evaluated using five classification issues and two cross-validation techniques: k-fold and leave-one-subject-out. The goal of these studies was to assess the proposed methodologies and identify the optimal combination strategy for establishing clinical decision support systems for the diagnosis and early detection of AD. The results of the suggested diagnostic system are presented and further explored below in the subsequent sections.

The remainder of this study is organized as follows: Section 2 describes the utilized dataset, pre-processing, feature extraction, and classification algorithms. Section 3 is dedicated to presenting the results and discussing them. The advantages and benefits have been discussed in section 4. Section 5 has been dedicated to discussing the limits, and future research prospects. Lastly, Section 6 is dedicated to presenting the conclusions.

## 2. Materials and methods

**Figure 1:**
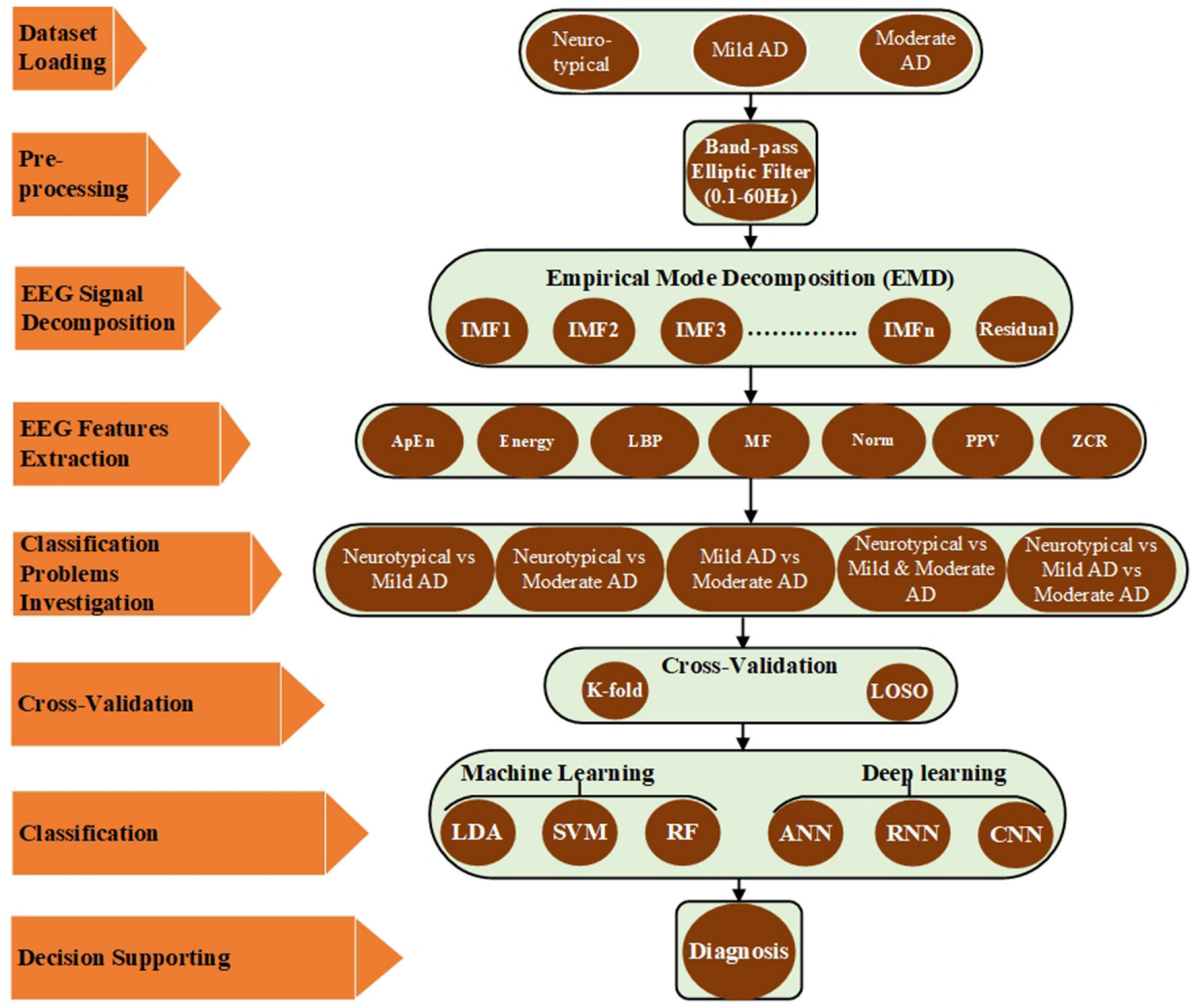
Block diagram of the proposed method based on EMD.

This section provides a detailed account of the employed EEG dataset as well as the proposed pre-processing, feature extraction, and classification methods. MATLAB simulation tools were utilized to validate these approaches. Figure 1 illustrates the proposed approaches, which involved reading different EEG datasets (neurotypical, mild AD, and moderate AD) and using a band-pass filter to reduce noise and interference and improve the signal-to-noise ratio (SNR). The filtered EEG signal was then processed using the EMD algorithm to decompose the signal into its features, and the feature vectors were obtained by computing various signal features for EMD outputs, including approximate entropy (ApEn), signal energy (Energy), logarithmic band power (LBP), mean frequency (MF), Norm (Norm), peak-to-peak value (PPV), and zero crossing rate (ZCR). These features were then utilized to address five classification issues, and each issue has been evaluated by k-fold and LOSO cross-validation techniques. Finally, different machine learning techniques such as LDA, SVM, and RF and deep learning approaches such as ANN, RNN, and CNN have been used to help the neurologists in support the diagnosis decision. All possible combinations of the proposed methodologies were implemented and validated using MATLAB simulation tools. Each stage, from data description to the classification procedure, is discussed in further detail in the subsequent subparts.

### 2.1. Description of the dataset

#### 2.1.1. Participants

The datasets of AD patients and neurotypical participants for this investigation were collected by the Behavioral and Cognitive Neurology Section of the Department of Neurology and the Reference Center for Cognitive Disorders at the Hospital das Clinicas in São Paulo, Brazil. All AD patients and neurotypical participants were diagnosed, and the datasets were recorded by expert neurologists using the Brazilian version of the Clinical Dementia Rating (CDR) scale and the Mini-Mental State Examination (MMSE) (Brucki et al., 2003). The multi-channel EEG datasets were collected from 86 participants, divided into three groups. The first group consists of 35 neurotypical participants (NP), 16 men and 19 women (mean age 66.89 years, 8.18 SD). The inclusion criteria for the cognitively normal group were a CDR score of 0 and an MMSE ≥ 25, with a mean MMSE of 28 and a standard deviation of 2.2, as well as no sign of functional cognitive deterioration before enrollment based on an interview with the participants. The second group includes 31 mild-AD patients based on NINCDS-ADRDA (McKhann et al., 1984) and DSM-IVTR (Association et al., 1996) criteria, including 12 men and 19 women (mean age 75.23 years, 5.55 SD). Additional inclusion criteria for mild AD patients were 0.5 ≤ CDR ≤ 1 and MMSE ≤ 24, with a mean MMSE of 19.48 and a standard deviation of 3.16. The third group consists of 22 moderate AD patients (DSM-IV-TR), seven men and 15 women (mean age 73.77 years, 10.16 SD). The inclusion criteria for moderate AD patients were a CDR score of 2 and an MMSE score of ≤ 20, with a mean MMSE of 14.18 and a standard deviation of 3.69. An additional criterion for inclusion in both AD cohorts (AD1 and AD2) was the existence of functional and cognitive deterioration during the previous twelve months, as determined by a lengthy interview with a qualified informant. Diabetes mellitus, kidney illness, thyroid disease, alcoholism, liver disease, lung disease, or vitamin B12 insufficiency were also examined in both AD groups (Fraga et al., 2013). The datasets wrer obtained from 1,426, 1,514, and 930 trials for neurotypical, Mild AD, and Moderate AD participants, respectively. The datasets were recorded for an estimated duration of 11,408, 12,112, and 7,440 s for neurotypical, mild AD, and moderate AD participants, respectively. Table 1 summarized the statistical description of participants' characteristics.

**Table 1:** The statistical description of participants characteristics.

**Participants characteristics**	**Neurotypical**	**Mild AD**	**Moderate AD**
No. of participants	35	31	20
Age	66.89 (52–83)	75.23 (63–89)	73.77 (48–87)
Gender (M:F)	16:19	12:19	7:13
Education level (years)	8.77 (2–26)	4.81 (0–11)	4.73 (0–15)
Mini-mental state examination	28 (20–31)	19.48 (14–24)	14.18 (4–20)
Clinical dementia rating	0	≥0.5& ≤ 1	2
No. of windows	1,426	1,514	930
Period (s)	11,408	12,112	7,440

**Figure 2:**
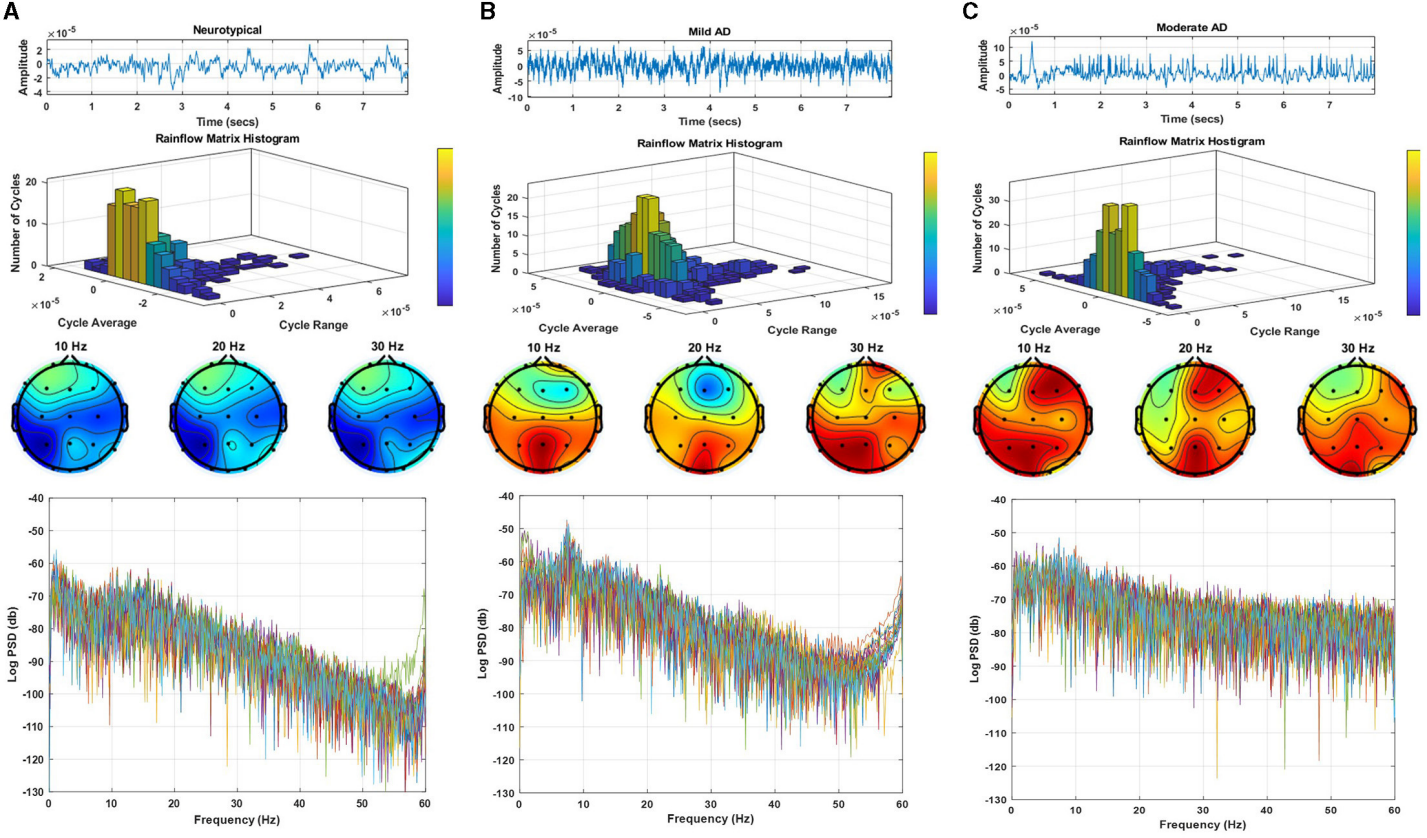
EEG signals sample, rainflow matrix histogram, electrodes maps, and power spectrum density pattern for **(A)** neurotypical EEG, **(B)** mild AD EEG, and **(C)** moderate AD EEG.

Figure 2 displays a sample of EEG signals, rainflow counting matrix, electrode mappings, and EEG power spectrum density with a logarithmic scale for three distinct datasets: neurotypical EEG (Figure 2A), Mild AD EEG (Figure 2B), and Moderate AD EEG (Figure 2C). The sample of EEG signals shows the recording from the Fp1 electrode of three random subjects from each of the three datasets. The rainflow counting matrix illustrates the number of cycles extracted from the EEG signal, and the plot of the rainflow matrix shows the number, range, and average of cycles. The electrode mappings are presented for three arbitrary frequencies: 10, 20, and 30 Hz, to highlight the differences between the three datasets. The distribution of EEG power across the EEG band is represented by the power spectrum density pattern. Generally, the power density of the low-frequency spectrum is greater than that of the high-frequency spectrum. By comparing the EEG signals of three different subjects, differences in amplitudes, rainflow plots, electrode mappings, and power spectrum density patterns can be observed.

#### 2.1.2. Data acquisition system

The Braintech 3.0 instrumentation collection system (EMSA Medical Equipments Inc., Brazil) captured the EEG dataset at a sample rate of 200 Hz and 12 bits of resolution. The International 10-20 System was used to install the electrodes for the EEG data-collecting device. Twenty electrodes—Fp1, Fp2, F3, F4, F7, F8, C3, C4, T3, T4, P3, P4, T5, T6—and two electrodes—A1 and A2 on the subject's left and right earlobes, respectively, were used to collect the EEG dataset for this study. The participants were awake, at ease, and had their eyes closed during the recording. EEG artifacts, such as blinking and muscle movements, were manually eliminated from the data by two expert neurophysiologists. Thereafter, 28 eight-second epochs from each EEG signal were chosen by eye inspection (Fraga et al., 2013).

### 2.2. Pre-processing

The artifacts, disturbances, and interferences were also recorded together with the EEG dataset. These artifacts, sounds, and interferences were produced by the electrodes, magnetic fields of electronics, blood pressure, breathing, limb movements, eye blinking, or other movements of human subjects (Müller-Gerking et al., 1999). The interferences and noises created during the EEG recording have been eliminated at the preprocessing stage by filtering the EEG signals using a band-pass filter. There have been numerous applications of infinite impulse response (IIR) and finite impulse response (FIR) filters. This study has looked at the band passes of an IIR elliptic digital filter with cutoff frequencies of 0.1 and 60 Hz. Two knowledgeable neurophysiologists carefully eliminated the EEG artifacts (such as blinking and muscle movements) from the EEG recordings (Fraga et al., 2013).

### 2.3. EEG signal decomposition

For signal processing, especially with regards to EEG signals and other biological signals, the feature extraction stage is crucial for achieving the optimum results. To evaluate and break down the EEG signal into its component features, a variety of feature extraction techniques are used. A well-liked and widely utilized method, EMD, was employed in the current investigation. EMD is an adaptive technique for time series analysis. It was created to be practical and appropriate for studying time series that are complicated, non-stationary, and non-linear and contain a variety of simple inherent oscillations. Using empirical analysis, these inherent oscillatory patterns are found. The features of the data time scales are used to deconstruct the time series data. It is possible to reduce the majority of signal oscillations between a signal's local minima and maxima points without a zero crossover. In an iterative procedure known as the sifting process, the EMD algorithm divides the signal *x*(*t*) into non-overlapping time scale components termed Intrinsic Mode Functions (*IMFs*) and a residual (Zeiler et al., 2010). *IMFs* have the following qualities: A single local minima and maxima point, with a maximum one-point difference, separates two following zero crossings in an *IMF*. The lower and upper *IMF* envelopes at each position have an average that is zero. The EEG signal *x*(*t*) has been split into *i* Intrinsic Mode Functions *IMF*_*i*_(*t*) and residual function *R*_*n*_(*t*) throughout the sifting process, as shown by the subsequent steps (Huang et al., 1998; Wu and Huang, 2009; Zheng et al., 2014):

Step 1: Finding all local maximum and minimum points in the EEG signal *x*(*t*).
Step 2: Using cubic splines to create the higher *E*_+_(*t*) and lower *E*_−_(*t*) envelopes from the local extremum points.
Step 3: Using the following equation, compute the mean *M*_*i*_(*t*) envelope for the *jth* iteration

(1) $$\begin{array}{l} M_{\left(i,j\right)}\left(t\right)=\frac{E_{+}\left(t\right)+E_{-}\left(t\right)}{2} \end{array}$$

Step 4: Calculating the difference between the main signal and the mean envelope *M*_(*i, j*)_(*t*)

(2) $$\begin{array}{l} IMF_{i}\left(t\right)=IMF_{\left(i-1\right)}\left(t\right)-M_{\left(i,j\right)}\left(t\right) \end{array}$$

Where *IMF*_(*i*−1)_(*t*) = *X*(*t*) for the first iteration. Steps 1–4 are performed with the new value of *IMF*_*i*_(*t*) and the result *IMF*_*i*_(*t*) has been tested until the *IMF* conditions are satisfied.

Step 5: Subtract the *ith* *IMF* from the previous residual signal to obtain the new residual signal.

(3) $$\begin{array}{l} R_{i}\left(t\right)=R_{\left(i-1\right)}\left(t\right)-IMF_{i}\left(t\right) \end{array}$$

Step 6: Calculating N intrinsic mode functions for the remaining signal *R*_*i*_(*t*) as the main signal, all the above steps are repeated *N* times. In this case, the original signal is represented as:

(4) $$\begin{array}{l} X\left(t\right)=\overset{N}{\underset{i=1}{\sum }}IMF_{i}\left(t\right)+R_{N}\left(t\right) \end{array}$$

**Figure 3:**
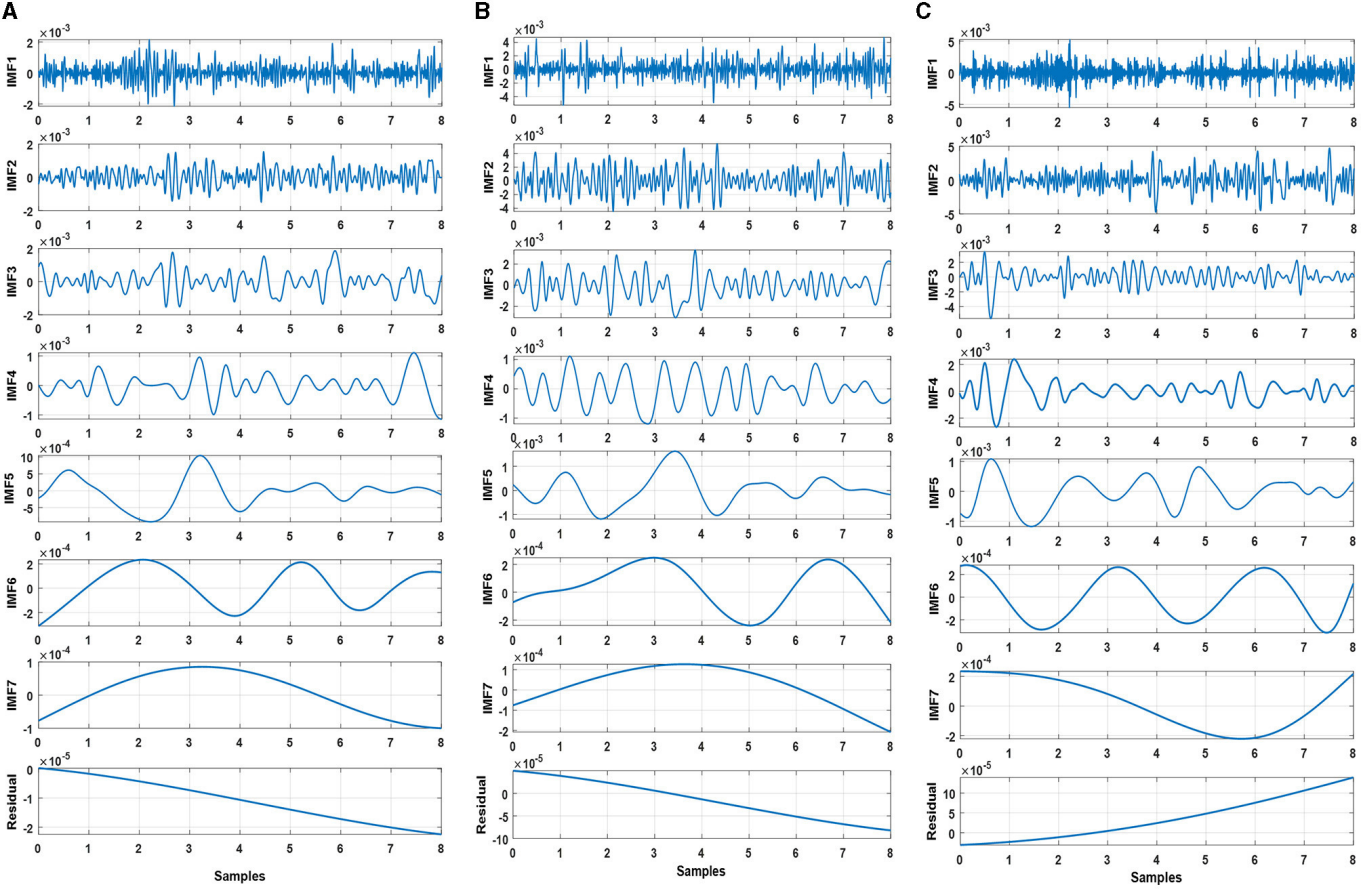
Decomposed intrinsic mode functions and residual for **(A)** neurotypical EEG, **(B)** mild AD EEG, and **(C)** moderate AD EEG.

The study employed EMD to process filtered EEG signals to extract the features of the signals. For a specific trial, EMD is applied to the recorded signal from the first electrode to decompose the signal into eight components, consisting of seven IMFs and residual components. This process is repeated for all the recorded signals from all electrodes, then for all trials with one participant, and then for all participants in the three groups (neurotypical, mild AD, and moderate AD). Figure 3 displays the Extracted IMFs and residuals by the EMD technique as an example of the decomposition of one of the trials for the Fp1 electrode from three different EEG classes (neurotypical, mild AD, and moderate AD). Figure 3A displays the IMFs and residual extracted from the neurotypical EEG signal, while Figure 3B shows those extracted from the Mild AD EEG signal, and Figure 3C displays those extracted from the Moderate AD EEG signal. As noted, the frequency and amplitude contents of the displayed IMFs and residual decreased from the first IMF to the seventh IMF and the residual component. For a thorough understanding of EMD outputs, it is essential to investigate some features, including their spectral, magnitude, and statistical features. Appendix 1 presents a summary of some spectral, magnitude, and statistical details for the displayed IMFs and residuals.

### 2.4. Feature extraction

In this study, seven significant features (parameters) were combined with the EMD technique to generate feature vectors and improve AD system performance diagnosis. To construct the feature vectors, the significant features were computed for all extracted components, which included both the IMFs and the residual, from all signals of a certain trial. These feature vectors were then processed individually through the classifiers in order to reduce the proposed system complexity and simplify the training process. The performance of each classifier has been evaluated for each significant feature (parameter). This will also be discussed in Section 3. This part provides a brief discussion of selected significant signal aspects as well as the rationale for their use in signal analysis. The feature vectors were constructed using the following significant features for a given discrete signal *x*(*n*) with mean μ and standard deviation σ, where *n* = 1,2,...., *N*, and *N* is the number of signal samples; the feature vectors were formed using the following significant features:

- **Approximate entropy (ApEn)**
  Approximate entropy (ApEn) is a pattern-based approach for estimating the regularity of a set of discrete-time signals. ApEn's computation seeks to estimate the randomness of a series of discrete time signals without prior knowledge of the source providing the dataset. As a result, ApEn can be calculated as follows:

(5) $$\begin{array}{l} X_{ApEn(e,r,N)}=\frac{1}{N-e+1}\sum_{i-1}^{N-e+1}log\\ \;C_{i}^{e}(r)-\frac{1}{N-e}\sum_{i-1}^{N-e}logC_{i}^{e+1}(r) \end{array}$$

where a series of patterns of length *e* (called the embedding dimension, which is the smallest integer for which the patterns do not intersect with each other) is derived from *x*(*n*), the index *r* is a fixed parameter that sets the tolerance of the comparison, and $C_{i}^{e}\left(r\right)$ is the correlation integral. The Correlation integral is given by:

(6) $$\begin{array}{l} C_{i}^{e}\left(r\right)=\frac{1}{N-e+1}\overset{N-e+1}{\underset{j-1}{\sum }}\phi \left(r-||X_{i}-X_{j}||\right) \end{array}$$

where *X*_*i*_, *X*_*j*_ are the points of the trajectory in the phase space, *r* is the radial distance around each reference point *X*_*p*_ and ϕ() is the Heaviside function (Acharya et al., 2012).

- **Energy (Energy)**
  Estimating the total energy of the EEG signal is critical for EEG signal classification. The energy of an EEG signal is defined as the sum of the signal's squared magnitude. This statistic is used to calculate the amount of energy dispersed over the signal. The EEG signal is complex, non-linear, and non-stationary, with information in both the time and frequency domains. With machine-learning systems, the assessment of total energy dispersed across the EEG signal may be a helpful indicator (Boashash et al., 2016).

(7) $$\begin{array}{l} X_{Energy}=\overset{N}{\underset{n=1}{\sum }}|x\left(n\right){|}^{2} \end{array}$$

- **Logarithmic band power (LBP)**
  In EEG spectrum analysis, it is usual to take the magnitude-squared of the EEG signal's absolute value, denoted by (μV), or the logarithm of the magnitude-squared of the EEG signal's absolute value, denoted by (db). The logarithmic band power is composed of variations in EEG frequency band activities at various periods of life that can summarize the frequency band's contribution to the overall power of the signal. This contribution may be valuable in machine-learning methodologies and may be the key parameter for AD researchers to differentiate between the various stages of AD (Xiao et al., 2018).

(8) $$X_{LBP}=\log(\frac{1}{N}\sum_{n=1}^{N}|x(n){|}^{2}$$

- **Mean frequency (MF)**
  The mean frequency is an average frequency, which is defined as the sum of the product of the signal power spectrum and the frequency divided by the total sum of the power spectrum. In addition, the mean frequency is also referred to as mean power frequency and mean spectral frequency in several works. The following is a definition of mean frequency:

(9) $$\begin{array}{l} X_{MF}=\frac{\sum_{j=1}^{M}f_{j}P_{j}}{\sum_{j=1}^{M}P_{j}} \end{array}$$

where *f*_*j*_ is the frequency value of the signal power spectrum at the frequency bin *j*, *P*_*j*_ is the signal power spectrum at the frequency bin *j*, and *M* is the length of frequency bin (Phinyomark et al., 2012).

- **Norm (Norm)**
  Norm is one of the most commonly encountered concepts for signal energy and intensity. When the strength of EEG signals varies in relation to AD phases, the norm is defined as the square root of the sum of the squares of the signal. As a result, the signal norm may be a valuable machine and deep-learning statistic (Knott et al., 2000).

(10) $$\begin{array}{l} X_{Norm}=\sqrt{\overset{N}{\underset{n=1}{\sum }}|x\left(n\right){|}^{2}} \end{array}$$

- **Peak-to-peak value (PPV)**
  Peak-to-peak amplitude is the difference between the highest and lowest values in a waveform. It is also a measure of the dispersion of outcomes, known in statistics as the range (Knopp, 2013).

(11) $$\begin{array}{l} X_{PPV}=|max\left(x\left(n\right)\right)-min\left(x\left(n\right)\right)| \end{array}$$

- **Zero-crossing rate (ZCR)**
  In the context of discrete-time signals, a zero crossing is said to occur when successive samples have different algebraic signs. It is the number of sign shifts between successive signal values that are positive to zero to negative or negative to zero to positive, divided by the total number of values. Zero crossing rates are an easy way to determine the frequency content of a signal. The zero-crossing rate is the number of times the amplitude of signals crosses through zero in a given time period or frame. It is feasible to get reasonable estimates of the spectrum properties of an EEG signal using a representation based on the zero-crossing rate (Chen, 1988).

(12) $$\begin{array}{l} X_{ZCR}=\frac{1}{N}\overset{N}{\underset{n=1}{\sum }}|sgn\left(x\left(n\right)\right)-sgn\left(x\left(n-1\right)\right)| \end{array}$$

where sgn(.) is the sign function, i.e.,

(13) $$sgn(x(n))=\{\begin{array}{ll} 1, & \text{if }x(n)\geq \text{0}\\ -1, & \text{otherwise} \end{array}$$

### 2.5. Cross-validation and classification

Before beginning the classification process, two separate cross-validation techniques were used to assess the robustness of the proposed approaches: k-fold and LOSO cross-validation. k-fold and LOSO cross-validation are two different techniques used to estimate the generalization performance of a machine learning model. Despite their differences, both techniques share the common goal of evaluating a model's ability to generalize to new data. As such, comparing a model's performance using both techniques can provide valuable insights into its generalization ability, identify potential issues with the model, and provide guidance on how to improve it under different conditions. For instance, if a model performs poorly on leave-one-subject-out cross-validation, it may indicate that the model is overfitting to the training data and is not able to generalize well to new subjects. To address this, one could consider collecting more data or improving the model's ability to generalize to new subjects. If a model performs poorly on k-fold cross-validation, it may indicate that the model is not able to capture the variability in the data due to random sampling and may not generalize well to new (unseen) data. In this case, using more advanced regularization techniques or increasing the model's complexity could be considered. If a model performs well on both techniques, it suggests that the model is robust and can generalize well to new data and new subjects.

#### 2.5.1. K-fold cross-validation

The k-fold cross-validation technique randomly divides all EEG features into k-equal subsets. One subset is chosen for testing, while the others are used for training. This strategy has been tested k times (k-fold), with each subgroup tested once (Refaeilzadeh et al., 2009). In this study, we employed 10-fold cross-validation to load all of the EEG signal features from the feature vector generated by the feature extraction techniques and submit them to the 10-fold cross-validation. Following that, these features were divided into a 90% subset for training and a 10% subset for testing. Each time, a vector was sent into the testing classifier. The test classifier result was then validated using the cross-validation technique by comparing it to the state of the original test features. This procedure was repeated ten times, one vector being placed into the testing classifier each time. Finally, the results were averaged to get one overall diagnostic accuracy.

#### 2.5.2. Leave-one-subject-out cross-validation

This method is used to evaluate machine learning algorithms when they are used to make predictions on data that was not used to train the model (Vehtari et al., 2017). LOSO cross-validation was utilized in this investigation, one person was excluded in the first iteration. Except for the features taken from the left subject, which are used for testing, all features extracted from all subjects are used for training. The cross-validation technique then validated the test classifier result by comparing it to the state of the original test features. Another subject was left out for testing in the second iteration, and this process was repeated *N* times, where *N* is the number of participants left out. In the current study, 20% of the participants in each group were left out for testing. One vector was sent to the testing classifier each time. The findings were then averaged to provide a single overall diagnostic accuracy. The classification procedure was used after the cross-validation technique.

Various classifiers were employed and analyzed to achieve the best classification accuracy and diagnosis performance. The following techniques were utilized in this study: linear discrimination analysis (LDA), support vector machine (SVM), random forest (RF), artificial neural network (ANN), recurrent neural network (RNN), and convolutional neural network (CNN). In the current study, the diagnosis system has been evaluated using three key evaluation metrics: classifier precision, recall, and accuracy. The formulas used to calculate these essential evaluation metrics are as follows Raschka (2015):


(14) $$\begin{array}{l} Precision\%=\frac{TP}{TP+FP}*100 \end{array}$$

(15) $$\begin{array}{l} Recall\%=\frac{TP}{TP+FN}*100 \end{array}$$

(16) $$\begin{array}{l} Accuracy\%=\frac{TP+TN}{TP+FP+TN+FN}*100 \end{array}$$

where TP, FP denote the number of true and false positive diagnoses that mean EEG features extracted from class I are correctly and incorrectly diagnosed, respectively, and TN, FN denote the number of true and false negative diagnoses that mean EEG features extracted from class II are correctly and incorrectly diagnosed, respectively. Table 2 describes the utilized parameters in the classification techniques.

**Table 2:** The parameters description of the classifiers.

**Classifier**	**Parameters**
LDA	Discriminant type: “linear”, prior probabilities: “empirical”, score transformation: “none”
SVM	Kernel function: “linear”, cache size: “1000”, prior probabilities: “empirical”, score transformation: “none”, number of iteration: “1000”, method: “least square”
RF	Method: “AdaBoostM”, prior probabilities: “empirical”, learner: “decision tree”, learning rate: “0.01”, no. of learners: “100”
ANN	No. of hidden layers: “1”, no. of nodes: “10”, hideen layer transfer function: “logsig”, output layer transfer function: “softmax”, performance function: “mae”, train function: “trainbr”, no. of epochs: “100”, learning rate: “0.01”
RNN	Layer delays, “1:2”, no. of hidden layers: “1”, no. of nodes: “10”, hidden layer transfer function: “tanh”, output layer transfer function: “linear”, performance function: “mae”, train function: “trainbr”, no. of epochs: “100”, learning rate: “0.01”
CNN	Convolutional layers: “2”, dropout layers: “2”, batch normalization layers: “2”, leaky relu layers: “2”, fully connected layers: “2”, dropout value: “0.25”, filter size: “11*11”, no. of filters: “96”, minimum batch size: “64”, no. of epochs: “100”, softmax layer: “1”, output layer=“classification”, learning function: “adam”, schedule: “piecewise”, learning rate drop period: “125”, learn rate drop factor: “0.2”

## 3. Results and discussion

As previously stated, the EEG datasets used in this investigation were separated into three groups. The EEG datasets were collected from 35 neurotypical participants, 31 mild AD patients, and 22 moderate AD patients in the first, second, and third groups, respectively. The EEG datasets were filtered using a band-pass elliptic filter with cut-off frequencies of 0.1 and 60 Hz to remove noise and increase the signal-to-noise ratio. Following that, the filtered signal was fed into the EMD approach to decompose the EEG-filtered signal into its features (IMFs and residual). The EMD technique was then integrated with a variety of signal properties such as ApEn, energy, LBP, etc. to create EEG feature vectors and increase diagnosis performance. Ultimately, multiple types of classifiers were used to discriminate EEG features belonging to their classes, and the classification accuracy was computed and compared. Two alternative cross-validation techniques, k-fold and LOSO cross-validation, were employed to further evaluate the proposed methodologies. The classification accuracy demonstrates the classifier's capacity to distinguish between neurotypical and AD subjects, AD stage patients and neurotypical subjects, and mild and moderate AD patients. Five classification issues have been examined based on the number of EEG dataset groups, as follows:

1. Neurotypical vs. mild AD features (2-class)
2. Neurotypical vs. moderate AD features (2-class)
3. Mild AD vs. moderate AD features (2-class)
4. Neurotypical vs. mild AD and moderate AD features (2-class)
5. Neurotypical vs. mild AD vs. moderate AD features (3-class)

The classification results for the five classification issues have been presented from Sections 3.1–3.5.

### 3.1. Neurotypical vs. mild AD features (2-class)

In this part, the EEG features extracted from 35 neurotypical subjects (class: “Neurotypical”) have been joined with the EEG features extracted from 31 mild AD patients (class: “mild AD”). The result of this combination forms the first classification issue (neurotypical vs. mild AD). The proposed approaches' performance was assessed using 10-fold and LOSO cross-validation techniques.

Tables 3, 4 present the total average classification precision, recall, and accuracy of six classifiers for the first classification issue based on EMD outputs and different signal feature combinations, using 10-fold and LOSO cross-validation approaches, respectively.

**Table 3:** Classification precision, recall, and accuracy for neurotypical vs. mild AD features based on k-fold cross-validation techniques.

	**LDA**			**SVM**			**RF**			**ANN**			**RNN**			**CNN**		
**Features**	**Pre**.	**Rec**.	**Acc**.	**Pre**.	**Rec**.	**Acc**.	**Pre**.	**Rec**.	**Acc**.	**Pre**.	**Rec**.	**Acc**.	**Pre**.	**Rec**.	**Acc**.	**Pre**.	**Rec**.	**Acc**.
EMD + ApEn	89.1	77.1	83.3	86.8	78.1	82.6	91.0	81.9	86.5	95.7	91.5	93.5	97.7	94.1	95.8	99.7	99.1	99.4
EMD + energy	89.2	77.2	83.4	57.7	52.8	55.7	95.8	91.6	93.6	85.4	77.2	81.4	92.3	84.5	88.4	94.9	90.7	92.7
EMD + LBP	**94.7**	**84.7**	**89.7**	**95.1**	**86.7**	**90.8**	96.1	91.9	93.9	93.7	89.8	91.6	**99.9**	**99.5**	**99.7**	**99.9**	**99.7**	**99.8**
EMD + MF	86.3	74.7	80.9	86.6	77.3	82.2	92.2	84.2	88.2	**96.8**	**92.8**	**94.7**	99.4	96.0	97.6	99.9	99.4	99.6
EMD + norm	91.5	82.0	86.8	80.6	77.0	78.6	**96.4**	**92.2**	**94.2**	96.8	92.6	94.6	99.1	92.6	97.3	99.7	99.0	99.3
EMD + PPV	87.2	78.1	82.8	54.0	52.3	52.5	93.3	89.3	91.2	88.9	80.2	84.6	95.7	91.5	93.5	95.6	91.4	93.4
EMD + ZCR	87.3	78.6	83.1	79.5	75.6	77.4	90.9	81.8	84.4	95.6	91.2	93.3	98.8	95.2	96.9	99.5	98.9	99.2

The bold values represent the highest classification precision, recall, and accuracy achieved by each classifier.

**Table 4:** Classification precision, recall, and accuracy for neurotypical vs. mild AD features based on LOSO cross-validation techniques.

	**LDA**			**SVM**			**RF**			**ANN**			**RNN**			**CNN**		
**Features**	**Pre**.	**Rec**.	**Acc**.	**Pre**.	**Rec**.	**Acc**.	**Pre**.	**Rec**.	**Acc**.	**Pre**.	**Rec**.	**Acc**.	**Pre**.	**Rec**.	**Acc**.	**Pre**.	**Rec**.	**Acc**.
EMD + ApEn	79.8	67.9	74.7	75.4	64.2	70.8	84.6	71.2	78.6	91.1	78.8	85.1	96.1	82.5	89.3	97.8	88.7	93.2
EMD + energy	78.3	65.9	73.1	60.9	52.7	58.3	92.1	77.2	84.8	78.7	68.8	74.4	88.2	74.3	81.7	91.3	77.1	84.6
EMD + LBP	**86.9**	**72.8**	**80.3**	**88.4**	**75.8**	**82.6**	94.2	80.1	87.2	91.3	77.1	84.6	**98.2**	**88.7**	**93.4**	98.2	89.4	93.7
EMD + MF	76.5	64.6	71.6	78.3	65.9	73.1	86.9	72.5	80.2	**94.2**	**80.5**	**87.4**	97.7	85.4	91.5	**98.2**	**89.7**	**93.9**
EMD + norm	84.3	70.9	78.3	76.1	65.2	71.6	**94.2**	**80.5**	**87.4**	94.1	79.8	87.1	97.7	84.8	91.2	97.8	88.6	93.1
EMD + PPV	79.5	66.9	74.1	53.7	50.3	52.2	90.9	75.8	83.7	82.1	71.5	77.3	91.6	79.5	85.7	91.2	78.8	85.2
EMD + ZCR	78.7	66.2	73.5	76.0	65.2	71.2	86.2	72.5	79.9	91.9	79.5	85.8	97.8	87.1	92.3	97.8	88.6	93.1

The bold values represent the highest classification precision, recall, and accuracy achieved by each classifier.

Based on k-fold and LOSO cross-validation procedures, Tables 3, 4 show that RF, ANN, RNN, and CNN classifiers achieve the highest classification accuracy. The proposed techniques attained an average accuracy of 94.2, 94.7, 99.7, and 99.8% based on k-fold and 87.4, 87.4, 93.4, and 93.9% based on LOSO. LBP, MF, and Norm are the retrieved features that provide the highest classification accuracy. When we compare our results in this part to those published in similar previous studies, we see that our approach produced greater diagnostic accuracy. Morabito et al. (2016) used a CNN classifier and attained a maximum accuracy of 85% utilizing the k-fold cross-validation technique. We achieved a maximum classification accuracy of 99.8% using EMD + LBP and CNN classifiers. Furthermore, we discover that our study outperforms (Ieracitano et al., 2020) in terms of classification accuracy. By combining a continuous wavelet transform with a bispectrum feature for feature extraction and a multi-layer perceptron classifier, Ieracitano et al. (2020) achieved a maximum accuracy of 96.24%. Our methods outperformed Fiscon et al. (2018a,b) investigations in terms of classification accuracy. Fiscon et al. (2018a,b) achieved 93.3% maximum classification accuracy by combining Fourier transform and WT feature extraction with the J48 classifier. Regarding this section, our study outperformed Pirrone et al. (2022) study. Their study used FRF and PCA for feature extraction and DT, SVM, and KNN for classification. Their study achieved a maximum diagnosis accuracy of 97% based on k-fold cross-validation.

### 3.2. Neurotypical vs. moderate AD features (2-class)

In this part, the EEG features extracted from 35 neurotypical subjects (class: “Neurotypical”) have been joined with the EEG features extracted from 22 moderate AD patients (class: “moderate AD”). The result of this combination forms the second classification issue (neurotypical vs. moderate AD). The performance of the proposed approaches has been assessed using 10-fold and LOSO cross-validation techniques. The average classification precision, recall, and accuracy of six classifiers for the second classification issue based on EMD outputs and different combinations of signal features are presented in Tables 5, 6. The classification performance was evaluated using 10-fold and LOSO cross-validation techniques.

**Table 5:** Classification precision, recall, and accuracy for neurotypical vs. Moderate AD features based on k-fold cross-validation techniques.

	**LDA**			**SVM**			**RF**			**ANN**			**RNN**			**CNN**		
**Features**	**Pre**.	**Rec**.	**Acc**.	**Pre**.	**Rec**.	**Acc**.	**Pre**.	**Rec**.	**Acc**.	**Pre**.	**Rec**.	**Acc**.	**Pre**.	**Rec**.	**Acc**.	**Pre**.	**Rec**.	**Acc**.
EMD + ApEn	87.0	80.1	87.4	87.2	80.1	87.5	93.4	88.3	92.9	98.4	95.4	97.6	98.9	97.3	98.5	99.8	99.5	99.7
EMD + energy	89.5	83.2	89.5	53.2	56.6	63.2	98.8	95.3	97.7	88.4	83.2	88.2	98.8	95.9	97.9	83.1	64.8	80.9
EMD + LBP	**96.7**	**93.4**	**96.1**	**97.4**	**94.4**	**96.8**	**99.0**	**95.5**	**97.8**	93.7	89.4	93.4	**99.8**	**99.5**	**99.7**	**99.9**	**99.8**	**99.9**
EMD + MF	90.1	83.4	89.9	91.6	85.4	91.1	95.3	91.1	94.7	98.2	95.1	97.4	99.0	97.7	98.7	**99.9**	**99.8**	**99.9**
EMD + norm	93.7	89.4	93.4	82.7	76.8	84.5	98.6	95.6	97.7	99.0	95.5	97.8	99.4	98.9	99.3	99.4	98.9	99.3
EMD + PPV	90.5	84.8	90.5	53.1	57.6	63.2	96.7	93.1	96.0	89.3	90.0	91.8	97.6	94.5	96.9	85.9	83.0	87.9
EMD + ZCR	92.8	87.5	92.4	86.9	79.7	87.2	96.3	92.4	95.6	**99.4**	**99.1**	**99.4**	99.1	98.8	99.2	99.7	99.4	99.6

The bold values represent the highest classification precision, recall, and accuracy achieved by each classifier.

**Table 6:** Classification precision, recall, and accuracy for neurotypical vs. moderate AD features based on LOSO cross-validation techniques.

	**LDA**			**SVM**			**RF**			**ANN**			**RNN**			**CNN**		
**Features**	**Pre**	**Rec**	**Acc**.	**Pre**.	**Rec**.	**Acc**.	**Pre**.	**Rec**.	**Acc**.	**Pre**.	**Rec**.	**Acc**.	**Pre**.	**Rec**.	**Acc**.	**Pre**.	**Rec**.	**Acc**.
EMD + ApEn	77.4	73.7	81.1	77.5	74.2	81.2	84.0	79.0	85.6	93.6	86.0	92.1	95.3	88.2	93.6	96.0	90.3	94.7
EMD + energy	79.4	74.7	82.3	47.2	59.7	57.7	**92.5**	**86.1**	**91.8**	89.0	66.1	83.3	94.7	87.1	92.9	72.9	56.5	74.5
EMD + LBP	**94.6**	**85.0**	**92.1**	**93.7**	**87.9**	**92.8**	**92.5**	**86.1**	**91.8**	92.9	77.4	88.7	**95.4**	**89.4**	**94.1**	**96.0**	**90.8**	**94.8**
EMD + MF	69.9	74.7	82.6	85.5	79.6	86.5	89.7	84.4	89.9	94.0	86.6	92.5	95.3	88.2	93.6	95.4	89.8	94.2
EMD + norm	85.6	80.1	86.8	85.3	56.5	78.9	92.0	86.0	91.5	94.1	86.6	92.6	**95.8**	**89.2**	**94.1**	94.8	88.7	93.6
EMD + PPV	83.9	78.5	85.5	47.7	60.8	58.1	90.8	85.0	90.6	89.8	66.7	83.9	94.2	87.1	92.8	94.1	87.1	92.7
EMD + ZCR	83.3	78.0	85.2	79.0	74.5	82.1	90.7	84.4	90.1	**99.9**	**89.8**	**94.3**	95.3	88.7	93.7	95.4	89.7	94.1

The bold values represent the highest classification precision, recall, and accuracy achieved by each classifier.

Tables 5, 6 show that the features classified by RF, ANN, RNN, and CNN classifiers produced the best results using k-fold and LOSO cross-validation procedures, respectively. The proposed approaches provided an average accuracy of 97.8, 99.4, 99.7, and 99.9% based on k-fold. According to LOSO, the best results were produced by ANN, RNN, and CNN classifiers, with average accuracy of 94.3, 94.1, and 94.8%. LBP, MF, and ZCR are the best features with the highest classification accuracy. When we compare our results to those of other research in this part, we discover that our study achieved an overall classification accuracy reach of 99.9% higher than the other studies using the 10-fold cross-validation technique. Morabito et al. (2016) used a CNN classifier to reach a maximum accuracy of 85%. By combining a continuous wavelet transform with a bispectrum feature for feature extraction and a multi-layer perceptron classifier, Ieracitano et al. (2020) achieved a maximum accuracy of 96.95%.

Triggiani et al. (2017) achieved a maximum diagnosis accuracy of 76.7% using Exact low-resolution brain electromagnetic tomography and an ANN classifier. Our methods outperformed Fiscon et al. (2018a,b) in terms of classification accuracy study. Their method attained a maximum classification accuracy of 80.6%. Simons et al. (2015) employed quadratic sample entropy for feature extraction and a linear discrimination analysis classifier to reach a maximum accuracy of 77.27% when using the LOSO cross-validation technique. In the study conducted by Houmani et al. (2018), epoch-based entropy and bump modeling were utilized to extract features from EEG signals, which were then classified using an SVM classifier. The proposed diagnosis system was 91.6% accurate. Regarding this section, our study outperformed Pirrone et al. (2022) study. Their study used FRF and PCA for feature extraction and DT, SVM, and KNN for classification. Their study achieved a maximum diagnosis accuracy of 96% based on k-fold cross-validation.

### 3.3. Mild AD vs. moderate AD features (2-class)

In this part, the EEG features extracted from 31 mild AD patients (class: “mild AD”) have been merged with the EEG features extracted from 22 moderate AD patients (class: “moderate AD”). The result of this combination is the third classification issue (mild AD vs. moderate AD). With the use of 10-fold and LOSO cross-validation methodologies, the performance of the suggested methods has been evaluated. Using EMD outputs and various signal features combinations, Tables 7, 8 provide the overall average classification precision, recall, and accuracy of six classifiers for the third classification issue.

**Table 7:** Classification precision, recall, and accuracy for mild AD vs. moderate AD features based on k-fold cross-validation techniques.

	**LDA**			**SVM**			**RF**			**ANN**			**RNN**			**CNN**		
**Features**	**Pre**.	**Rec**.	**Acc**.	**Pre**.	**Rec**.	**Acc**.	**Pre**.	**Rec**.	**Acc**.	**Pre**.	**Rec**.	**Acc**.	**Pre**.	**Rec**.	**Acc**.	**Pre**.	**Rec**.	**Acc**.
EMD + ApEn	83.6	88.8	82.2	83.8	89.0	82.5	86.7	92.0	86.3	92.5	93.9	91.5	92.3	93.1	90.9	93.1	96.0	93.1
EMD + energy	83.8	89.0	82.5	70.4	66.2	61.8	88.6	93.5	88.5	84.5	90.0	83.6	85.8	91.2	85.1	85.9	91.3	85.3
EMD + LBP	**87.6**	**93.0**	**87.5**	**88.4**	**94.8**	**88.5**	**88.6**	**93.8**	**88.7**	88.6	93.8	88.7	**93.2**	**95.8**	**93.1**	93.7	94.3	92.6
EMD + MF	84.2	89.7	83.2	84.4	89.9	83.4	87.2	92.3	86.8	88.3	93.7	88.4	92.2	92.9	90.7	94.5	93.9	92.8
EMD + norm	86.0	91.4	85.4	78.9	83.8	76.1	88.0	93.5	88.1	**93.8**	**93.6**	**92.2**	92.3	93.9	91.4	92.2	94.9	91.9
EMD + PPV	81.4	90.8	81.4	75.0	59.9	62.8	85.4	90.8	84.7	81.9	88.6	80.8	88.6	93.8	88.7	85.5	90.9	84.8
EMD + ZCR	84.7	90.2	83.8	78.5	91.6	79.3	87.0	92.2	86.7	93.3	93.3	91.7	92.5	93.9	91.5	**94.0**	**92.8**	**93.6**

The bold values represent the highest classification precision, recall, and accuracy achieved by each classifier.

**Table 8:** Classification precision, recall, and accuracy for mild AD vs. moderate AD features based on LOSO cross-validation techniques.

	**LDA**			**SVM**			**RF**			**ANN**			**RNN**			**CNN**		
**Features**	**Pre**.	**Rec**.	**Acc**.	**Pre**.	**Rec**.	**Acc**.	**Pre**.	**Rec**.	**Acc**.	**Pre**.	**Rec**.	**Acc**.	**Pre**.	**Rec**.	**Acc**.	**Pre**.	**Rec**.	**Acc**.
EMD + ApEn	85.9	72.9	75.8	80.3	78.2	74.6	79.6	88.1	78.7	85.4	91.4	85.1	85.0	91.7	84.9	**90.2**	**91.4**	**88.6**
EMD + energy	85.8	71.9	75.2	63.7	53.0	52.2	82.1	92.7	83.1	79.4	86.8	77.8	79.7	88.4	78.8	80.0	88.7	79.4
EMD + LBP	**81.1**	**90.7**	**81.1**	**81.9**	**92.7**	**82.8**	**82.3**	**92.7**	**83.3**	82.8	93.1	83.7	**86.7**	**92.7**	**86.7**	87.0	93.1	87.1
EMD + MF	79.3	86.4	77.6	79.4	86.8	77.8	77.8	78.7	79.2	82.6	92.7	83.4	85.3	91.5	84.9	87.3	93.1	87.4
EMD + norm	80.1	89.4	79.7	72.5	74.5	66.7	81.9	92.7	82.8	**86.0**	**91.7**	**85.6**	85.7	91.4	85.2	86.1	92.7	86.2
EMD + PPV	79.9	76.5	73.5	64.7	54.6	53.4	78.6	87.4	77.4	85.2	66.9	72.3	84.4	91.4	84.3	78.4	86.4	76.8
EMD + ZCR	79.3	87.4	78.1	84.5	66.9	71.9	79.9	89.4	79.6	85.6	91.8	85.3	85.4	91.4	85.1	**90.2**	**91.4**	**88.6**

The bold values represent the highest classification precision, recall, and accuracy achieved by each classifier.

According to Tables 7, 8, which were created using k-fold and LOSO cross-validation procedures, the features classified by ANN, RNN, and CNN classifiers produced higher results. The suggested methods, based on k-fold, had an average accuracy of 92.2, 93.1, and 93.6%, respectively. The best results, according to LOSO, were attained by ANN, RNN, and CNN classifiers, which had an average accuracy of 85.6, 86.7, and 88.6%, respectively. LBP, Norm, and ZCR are the top extracted features that offer the most accurate classification. When we compare the results from this part to those from previous research, we discover that our approach produced an overall classification accuracy that was 93.6% higher.

Morabito et al. (2016) used a CNN classifier and attained a maximum accuracy of 78% utilizing the k-fold cross-validation procedure. Ieracitano et al. (2020) used a continuous wavelet transform with a bispectrum feature for feature extraction and a multi-layer perceptron classifier to reach a maximum accuracy of 90.24%. Fiscon et al. (2018a,b) utilized the Fourier transform and WT feature extraction with the J48 classifier to get up to 66.7% classification accuracy. Amezquita-Sanchez et al. (2019) employed multiple signal identification, an empirical wavelet transform, and a probabilistic neural network model that was upgraded. The proposed method has a maximum diagnostic accuracy of 90.3%. Regarding this section, our study outperformed Pirrone et al. (2022) study. Their study used FRF and PCA for feature extraction and DT, SVM, and KNN for classification. Their study achieved a maximum diagnosis accuracy of 83% based on k-fold cross-validation.

### 3.4. Neurotypical vs. mild and moderate AD features (2-class)

In this part, the EEG features extracted from 35 neurotypical subjects (class: “Neurotypical”) have been merged with the EEG features extracted from both groups of 31 mild AD and 22 moderate AD patients (class: “mild & moderate AD”). The result of this combination is the fourth classification issue (neurotypical vs. mild & moderate AD). The suggested methodologies' performance was assessed using 10-fold and LOSO cross-validation techniques. Tables 9, 10 show the total average classification precision, recall, and accuracy of six classifiers for the fourth classification issue using EMD output and different feature combinations based on 10-fold and LOSO cross-validation approaches, respectively.

**Table 9:** Classification precision, recall, and accuracy for neurotypical vs. mild and moderate AD features based on k-fold cross-validation techniques.

	**LDA**			**SVM**			**RF**			**ANN**			**RNN**			**CNN**		
**Features**	**Pre**.	**Rec**.	**Acc**.	**Pre**.	**Rec**.	**Acc**.	**Pre**.	**Rec**.	**Acc**.	**Pre**.	**Rec**.	**Acc**.	**Pre**.	**Rec**.	**Acc**.	**Pre**.	**Rec**.	**Acc**.
EMD + ApEn	84.9	85.3	81.1	85.1	86.1	81.7	89.2	87.5	85.4	96.1	95.8	94.9	97.8	97.6	97.1	99.6	99.5	99.4
EMD + energy	83.3	84.1	79.3	82.7	51.6	62.6	**97.4**	**91.9**	**93.3**	90.2	83.6	83.9	94.9	92.0	91.8	96.3	91.7	92.3
EMD + LBP	**92.2**	**88.9**	**88.2**	**91.0**	**92.6**	**89.5**	**96.8**	**92.4**	**93.3**	92.4	89.2	88.5	**99.8**	**99.6**	**99.6**	**100**	**99.9**	**99.9**
EMD + MF	94.9	85.7	81.3	87.1	84.9	82.5	90.0	90.1	87.4	95.6	95.1	94.1	98.0	97.8	97.3	99.8	99.6	99.6
EMD + norm	89.6	88.9	86.5	79.2	78.1	73.2	**96.1**	**93.2**	**93.3**	**98.9**	**98.8**	**98.5**	98.6	98.5	98.1	99.8	99.7	99.7
EMD + PPV	85.9	85.4	81.9	82.7	52.8	63.2	92.6	92.4	90.5	88.2	86.5	84.1	96.8	92.4	93.3	96.1	95.5	94.7
EMD + ZCR	89.2	83.5	83.2	83.5	85.7	80.3	89.7	88.5	86.3	97.0	96.4	95.8	96.5	97.3	96.7	99.6	99.6	99.5

The bold values represent the highest classification precision, recall, and accuracy achieved by each classifier.

**Table 10:** Classification precision, recall, and accuracy for neurotypical vs. mild and moderate AD features based on LOSO cross-validation techniques.

	**LDA**			**SVM**			**RF**			**ANN**			**RNN**			**CNN**		
**Features**	**Pre**.	**Rec**.	**Acc**.	**Pre**.	**Rec**.	**Acc**.	**Pre**.	**Rec**.	**Acc**.	**Pre**.	**Rec**.	**Acc**.	**Pre**.	**Rec**.	**Acc**.	**Pre**.	**Rec**.	**Acc**.
EMD + ApEn	80.4	78.5	74.3	80.2	77.9	73.9	86.0	76.8	77.5	91.9	90.8	89.1	93.8	93.2	91.8	94.4	93.9	92.6
EMD + energy	79.4	77.5	73.1	65.9	55.1	53.8	90.8	90.6	88.2	82.2	80.3	76.7	88.3	86.7	84.3	88.8	87.7	85.3
EMD + LBP	**85.5**	**85.7**	**81.8**	**91.2**	**80.9**	**83.1**	**91.0**	**90.8**	**88.5**	86.0	85.7	82.2	**95.1**	**94.7**	**93.5**	**95.5**	**94.9**	**93.9**
EMD + MF	80.3	87.9	74.1	81.4	79.7	75.7	84.8	84.6	80.7	90.8	90.8	88.3	93.4	93.0	91.5	94.8	94.3	93.1
EMD + norm	84.3	84.6	80.4	85.0	63.1	69.8	90.8	90.8	88.4	**94.4**	**93.9**	**92.6**	94.4	94.1	92.8	95.1	94.5	93.4
EMD + PPV	80.4	78.3	74.2	66.3	55.9	54.3	88.3	86.3	84.1	82.0	80.1	76.4	89.2	89.3	86.4	91.3	90.8	88.7
EMD + ZCR	82.2	80.5	76.8	80.0	77.7	73.6	82.2	84.4	78.7	91.0	91.2	88.7	93.4	92.6	91.2	95.3	94.7	93.6

The bold values represent the highest classification precision, recall, and accuracy achieved by each classifier.

Tables 9, 10 show that the features classified by ANN, RNN, and CNN classifiers produced superior results using k-fold and LOSO cross-validation procedures, respectively. The proposed approaches obtained an average accuracy of 98.5, 99.6, and 99.9% based on k-fold. According to LOSO, the best results were produced by ANN, RNN, and CNN classifiers, with an average accuracy of 92.6, 93.5, and 93.9%, respectively. LBP and Norm are the best-extracted features that offer the highest classification accuracy. When we compare our results in this part to those of other research, we see that our work offered overall classification accuracy that was greater than that reported in previous studies, reaching 99.9 and 93.9% for 10-fold and LOSO cross-validation procedures, respectively. Cassani et al. (2017) employed an automated artifact removal approach utilizing common EEG features, spectral power, and coherence to extract the amplitude-modulation features using the k-fold cross-validation technique. To reach a maximum accuracy of 91.1%, use an SVM classifier.

Trambaiolli et al. (2017) employed Wavelet and visibility graphs for feature extraction with an SVM classifier to get up to 91.18% classification accuracy. Fiscon et al. (2018a,b) utilized the Fourier transform and WT feature extraction with the J48 classifier to get up to 84.4% classification accuracy. Kanda et al. (2014) employed a Morlet wavelet filter for feature extraction and an SVM algorithm for classification to achieve up to 83.95% classification accuracy. Cassani et al. (2017) reached a maximum accuracy of 81.4% using the LOSO cross-validation approach. Trambaiolli et al. (2017) reached up to 85.29% classification accuracy. Ruiz-Gómez et al. (2018b) extracted features using spectral and non-linear features and classified them using a multi-layer perceptron. This proposed method has a maximum accuracy of 78.43%. Also, our study outperformed Maturana-Candelas et al. (2019). Maturana-Candelas et al. (2019) achieved a maximum accuracy of 79.1% using MSE and rMSSE for feature extraction and a QDA classifier. Kanda et al. (2014) achieved an accuracy of up to 84.56% in classification. In another work, Cassani et al. (2014) estimated three EEG signal features: spectral, coherence, and amplitude modulation, and then utilized a SVM to obtain an accuracy of 84.7%. Upon comparing the outcomes of our study with those of Cura et al. (2021) study, we observed that our study outperformed theirs. Their study employed EMD, Ensemble EMD, and DWT for feature extraction and utilized DT, SVM, KNN, and RF for classification. Their study achieved a diagnostic accuracy of 96.5% based on k-fold cross-validation. Regarding this section, our study outperformed Pirrone et al. (2022) study. Their study used FRF and PCA for feature extraction and DT, SVM, and KNN for classification. Their study achieved a maximum diagnosis accuracy of 89% based on k-fold cross-validation. By comparing our study results with the result of Alessandrini et al. (2022) study, we find that our study outperformed theirs, which used RPCA and PCA for feature extraction and RNN technique for classification. Their study achieved a maximum diagnosis accuracy of 97.9% based on k-fold cross-validation.

### 3.5. Neurotypical vs. mild AD vs. moderate AD features (3-class)

In this part, the EEG features extracted from 35 neurotypical subjects (first class: “Neurotypical”) have been merged with the EEG features extracted from 31 mild AD patients (second class: “mild AD”) and merged with the EEG features extracted from 22 moderate AD patients (Third class: “moderate AD”) resulting in the fifth classification issue (neurotypical vs. mild AD vs. moderate AD). The suggested methodologies' performance was evaluated using 10-fold and LOSO cross-validation techniques. Tables 11, 12 show the total average classification precision, recall, and accuracy of six classifiers for the fifth classification issue using EMD output and different signal features using 10-fold and LOSO cross-validation approaches, respectively.

**Table 11:** Classification precision, recall, and accuracy for neurotypical vs. mild AD vs. moderate AD features based on k-fold cross-validation techniques.

	**LDA**			**SVM**			**RF**			**ANN**			**RNN**			**CNN**		
**Features**	**Pre**.	**Rec**.	**Acc**.	**Pre**.	**Rec**.	**Acc**.	**Pre**.	**Rec**.	**Acc**.	**Pre**.	**Rec**.	**Acc**.	**Pre**.	**Rec**.	**Acc**.	**Pre**.	**Rec**.	**Acc**.
EMD + ApEn	72.2	71.6	72.4	73.6	73.1	73.8	84.7	84.3	84.8	88.0	88.0	88.5	88.9	88.9	89.4	94.7	94.1	94.8
EMD + energy	73.2	72.6	73.4	51.9	52.2	52.5	**94.0**	**93.3**	**94.1**	74.0	72.9	74.1	82.6	82.1	82.7	89.3	89.2	89.8
EMD + LBP	**81.3**	**81.0**	**81.5**	**85.6**	**85.5**	**86.1**	93.3	93.1	93.8	80.0	87.6	80.1	**94.3**	**93.8**	**94.4**	94.7	95.1	95.2
EMD + MF	71.9	71.3	72.2	75.9	74.9	75.9	87.6	87.6	88.1	83.7	83.3	83.8	90.0	89.9	90.2	94.5	95.0	95.1
EMD + norm	79.5	77.1	78.5	66.5	66.7	67.6	**94.0**	**93.4**	**94.1**	**92.4**	**92.3**	**92.7**	91.8	91.8	92.1	94.6	95.1	95.2
EMD + PPV	72.1	71.6	72.4	50.3	50.7	50.7	91.5	91.3	91.6	74.1	73.1	74.2	86.3	86.0	86.6	90.3	90.0	90.4
EMD + ZCR	74.7	73.3	74.4	66.8	67.1	67.9	88.4	88.4	88.9	90.0	90.0	90.2	89.3	89.2	89.8	**95.2**	**95.7**	**95.7**

The bold values represent the highest classification precision, recall, and accuracy achieved by each classifier.

**Table 12:** Classification precision, recall, and accuracy for neurotypical vs. mild AD vs. moderate AD features based on LOSO cross-validation techniques.

	**LDA**			**SVM**			**RF**			**ANN**			**RNN**			**CNN**		
**Features**	**Pre**.	**Rec**.	**Acc**.	**Pre**.	**Rec**.	**Acc**.	**Pre**.	**Rec**.	**Acc**.	**Pre**.	**Rec**.	**Acc**.	**Pre**.	**Rec**.	**Acc**.	**Pre**.	**Rec**.	**Acc**.
EMD + ApEn	62.6	62.3	63.7	63.5	63.2	64.6	78.2	75.3	76.8	81.4	81.5	81.6	81.5	81.2	81.7	87.6	87.4	87.5
EMD + energy	64.6	64.3	65.9	44.0	44.0	44.9	85.2	85.1	85.2	64.5	64.2	65.8	75.3	72.6	74.3	82.2	82.1	82.4
EMD + LBP	**75.2**	**72.5**	**74.2**	**79.3**	**77.1**	**78.2**	85.6	85.5	85.6	73.2	72.5	73.4	**87.5**	**87.4**	**87.4**	**88.2**	**88.1**	**88.2**
EMD + MF	63.5	63.2	64.6	66.3	66.0	67.4	80.9	80.9	81.3	77.5	74.5	76.1	83.5	83.4	83.6	87.7	87.5	87.6
EMD + norm	72.1	70.7	71.9	57.1	57.1	58.7	**86.6**	**86.6**	**86.7**	**83.5**	**83.5**	**83.7**	85.1	84.9	85.1	87.4	87.2	87.3
EMD + PPV	61.5	61.3	62.7	40.6	40.6	41.1	83.5	83.3	83.5	64.5	64.2	65.8	80.3	78.8	79.3	83.0	82.8	83.2
EMD + ZCR	65.7	65.4	66.8	57.0	56.9	58.5	81.9	81.9	82.1	83.4	83.3	83.6	82.6	82.5	82.8	87.8	87.6	87.7

The bold values represent the highest classification precision, recall, and accuracy achieved by each classifier.

Based on k-fold and LOSO cross-validation procedures, Tables 11, 12 show that features classified by RF, ANN, RNN, and CNN classifiers produced better results. Based on the k-fold cross-validation, the proposed techniques obtained an average accuracy of 94.1, 92.7, 92.1, and 99.9% using RF, ANN, RNN, and CNN classifiers, respectively. According to LOSO, the best results were produced by RF, ANN, RNN, and CNN classifiers, which reached an average accuracy of 86.7, 83.7, 87.4, and 88.2%, respectively. LBP and Norm are the extracted features that offer the maximum classification accuracy. When we compare our results in this part to those of other research, we find that our work produced greater overall classification accuracy than those published in previous publications, reaching 95.2 and 88.2% for 10-fold and LOSO cross-validation procedures, respectively. Morabito et al. (2016) used a CNN classifier and attained a maximum accuracy of 82% using a 10-fold cross-validation procedure. By combining a continuous wavelet transform with a bispectrum feature for feature extraction and a multi-layer perceptron classifier, Ieracitano et al. (2020) obtained a maximum accuracy of 89.22%. Our study achieved better results than Rodrigues et al. (2016) study in this aspect. The latter study employed DWT and surrogate DT, as demonstrated by k-fold cross-validation, where our study achieved a diagnostic accuracy of 95.7%, while Rodrigues et al. (2016) study achieved up to 95.45%. On the other hand, Rodrigues et al. (2016) study outperformed our study in LOSO cross-validation, where their study achieved a diagnostic accuracy of up to 94.89%. Upon combining our study with Safi and Safi (2021) work, we observed that our study outperformed their work based on LOSO cross-validation, while their study outperformed ours based on k-fold cross-validation. Safi and Safi (2021) work achieved a maximum diagnosis accuracy of 97.64 and 81.08% based on k-fold and LOSO cross-validation, respectively.

Finally, our study outperformed Pirrone et al. (2022) study, which utilized FRF and PCA for feature extraction, and DT, SVM, and KNN for classification. Their study achieved a diagnostic accuracy of 86% based on k-fold cross-validation.

**Figure 4:**
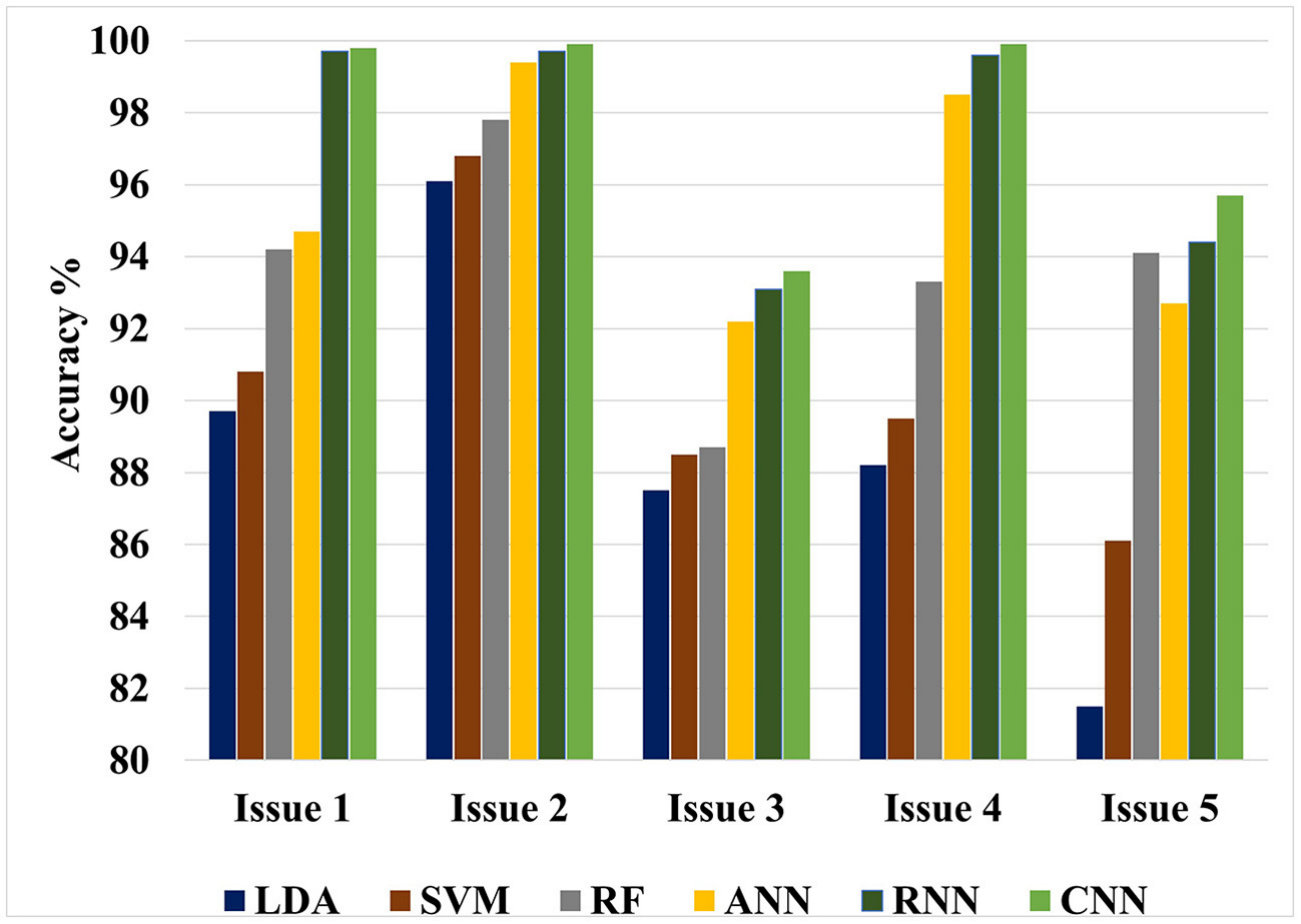
Maximum classification accuracy for all classification issues using all classifiers based on 10-fold cross-validation technique.

**Figure 5:**
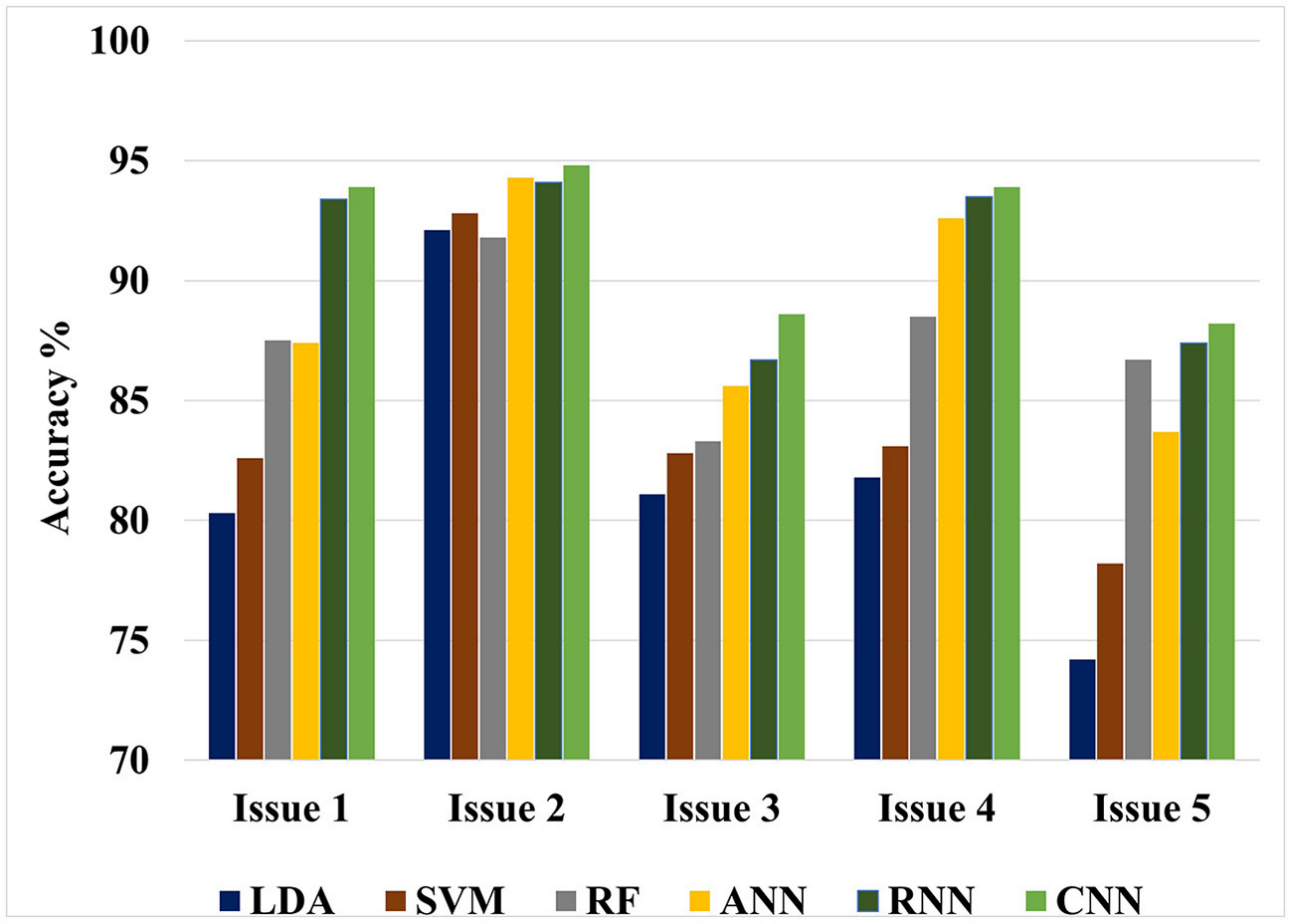
Maximum classification accuracy for all classification issues using all classifiers based on LOSO cross-validation technique.

The value of this study may be evaluated by comparing the findings of the suggested approaches to the results of other researches that have been done similarly. Figures 4, 5 show the maximum classification accuracy for all classification issues (issues 1–5) utilizing all classifiers using 10-fold and LOSO cross-validation approaches, respectively. Figures 4, 5 demonstrate that deep learning techniques, namely ANN, RNN, and CNN, achieved higher accuracy than machine learning techniques such as LDA, SVM, and RF in all classification issues except for the fifth issue, where RF outperformed ANN. Overall, RNN and CNN provided the highest accuracy in all five cases. A comparison of the classification issues reveals that the third and fifth issues yielded lower classification accuracy compared to the first, second, and fourth issues. This is likely due to the proposed system having difficulty classifying Alzheimer's signs with each other (mild AD and moderate AD) in the third case and also having difficulty distinguishing between three different types in the fifth case. Lastly, it is important to note that the classification accuracy based on LOSO was lower than that based on k-fold cross-validation because the proposed system was not trained on signals similar to those used in the test.

Appendix 2 presents the compression of our findings to those of prior studies on determining AD stages. Similar earlier investigations used EEG datasets for neurotypical, AD, and moderate cognitive impairment (MCI) datasets caused by early-stage AD (Mild AD), but omitted MCI datasets caused by other conditions.

## 4. Benefits of the study

The main benefits and advantages of the present work can be summarized as follows:

- Automatic diagnosis system: Development of an accurate clinical decision support system capable of automatically analyzing EEG signals to provide early diagnosis for Mild and Moderate AD.
- Evaluation variety: Evaluation of the proposed system using five classification issues and different cross-validation techniques, including k-fold and LOSO cross-validation techniques.
- Classification techniques variety: Use of two types of artificial intelligence techniques, including machine and deep-learning techniques, in the classification process.
- Promising accuracy: Promising results were obtained for two-class diagnosis (neurotypical vs. mild AD, neurotypical vs. moderate AD, mild AD vs. moderate AD, neurotypical vs. mild AD and moderate AD) with superior performance and greater accuracies than prior studies reported in the literature. Likewise, the results for the three-class diagnosis (neurotypical vs. mild vs. moderate AD) show higher performance than similar earlier research.
- Clinical benefit: The proposed method has clinical benefits as it can assist medical practitioners and clinicians in diagnosing AD automatically, swiftly, conveniently, efficiently, and accurately. The proposed solutions could potentially reduce the limited number of neurologists, reduce diagnostic time, and increase diagnosis accuracy.

## 5. Limitations and future work

Despite the fact that we demonstrated the use of our proposed methodologies, some constraints must be addressed:

- Dataset size: The size of the employed EEG biomedical dataset is relatively small, consisting of 35 neurotypical individuals, 31 mild AD subjects, and 22 moderate AD subjects. A larger, public dataset could be used to test the robustness and universality of the proposed method for EEG signal classification. Future research will include validation of this study with a larger dataset and adapting the methodology to include input signals from various EEG recorders.
- Severe AD dataset: The proposed approaches were not evaluated with a severe AD dataset. It would be beneficial to identify various subtypes of AD and analyze the system's performance on different AD forms, including severe AD.
- Deep learning hyperparameters: Deep learning algorithms require selecting several hyperparameters to learn the model, but achieving the ideal values of those parameters can be difficult. Therefore, automatic tuning techniques may be necessary at times.

## 6. Conclusion

The current work focuses on the creation of an accurate clinical decision support system for early AD diagnosis based on EEG signal processing. In this study, the EEG datasets were filtered using a band-pass filter and decomposed into their features using the EMD approach. The EMD technique was then used to generate feature vectors and increase diagnosis performance by combining EMD output with numerous signal features. Following that, machine and deep learning algorithms were examined and compared for identifying extracted EEG signal aspects of mild AD, moderate AD, and neurotypical cases in order to provide insights into future methods of early AD detection.

K-fold and LOSO cross-validation procedures were also examined as validation methods, and classification precision, recall, and accuracy were calculated for evaluating classifier performance. This study intends to compare the offered methodologies and identify the optimum combination method for the early detection of AD. Five classification issues were studied, and the proposed diagnosis system was evaluated in light of those issues. Table 13 presents the summary of the best results for AD diagnosis using the EMD technique for five classification issues. In summary, the study suggests that the most effective combination for developing a reliable and precise diagnosis system to assist in detecting AD is the integration of an elliptical filter for pre-processing, EMD with LBP or ZCR for feature extraction, and CNN for classification.

**Table 13:** A summary of the best results for AD diagnosis using the EMD technique for five classification issues.

**Issue**	**Feature**	**Classifier**	**CV**	**Precision %**	**Recall %**	**Accuracy %**
Issue I	LBP	CNN	k-fold	**99.9**	**99.7**	**99.8**
	MF		LOSO	**98.2**	**89.7**	**93.9**
Issue II	LBP& MF	CNN	k-fold	**99.9**	**99.8**	**99.9**
	LBP		LOSO	**96.0**	**90.8**	**94.8**
Issue III	ZCR	CNN	k-fold	**94.0**	**92.8**	**93.6**
	ApEn& ZCR		LOSO	**90.2**	**91.4**	**88.6**
Issue IV	LBP	CNN	k-fold	**100**	**99.9**	**99.9**
			LOSO	**95.5**	**94.9**	**93.9**
Issue V	ZCR	CNN	k-fold	**95.2**	**95.7**	**95.7**
	LBP		LOSO	**88.2**	**88.1**	**88.2**

The bold values represent the highest classification precision, recall, and accuracy achieved by each classifier.

## Data availability statement

The original contributions presented in the study are included in the article/Supplementary material, further inquiries can be directed to the corresponding author.

## Ethics statement

The studies involving human participants were reviewed and approved by Ethics Committee for the Analysis of Research Projects—CAPPesq of the Clinical Board of Hospital das Clinicas and of the Faculty of Medicine of the University of São Paulo (Application No. 0630/09). The patients/participants provided their written informed consent to participate in this study.

## Author contributions

AA: conceptualization and investigation. KA: data curation, formal analysis, methodology, software, and writing—original draft. FA: funding acquisition, project administration, and supervision. YS: resources. MA: validation, visualization, writing—review, and editing. All authors have read and agreed to the published version of the manuscript.
