# Supplementary material for: EEG-based clinical decision support system for Alzheimer's disorders diagnosis using EMD and deep learning techniques
Source: Front Hum Neurosci. 2023 Aug 31;17:1190203. doi: 10.3389/fnhum.2023.1190203 (PMC10501399; doi:10.3389/fnhum.2023.1190203)
Supplement: Supplementary file 1 [file Table_1.docx]

**APPENDIX 1**

In the study, the EMD technique was used to decompose each signal into eight vectors (IMF1-IMF7 and residual). Figure 3 displays the generated IMFs and residual by the EMD technique as an example of the decomposition of one of the trials for the Fp1 electrode from three different EEG datasets (Neurotypical, Mild AD, and Moderate AD). In this section, we have computed some spectral, magnitude, and statistical contents of the displayed IMFs and residual to provide a comprehensive understanding of the outputs obtained from EMD. These computations allow for a detailed analysis of the EMD outputs and provide valuable insights into the features of the signal being analyzed. The computed contents consist of three categories: spectral, magnitude, and statistical. The spectral contents include the spectral centroid (SpCe), bandwidth (SpBw), and flatness (SpFt). The magnitude contents comprise ApEn, ZCR, and crest factor (CF). Lastly, the statistical contents consist of mean (M), standard deviation (SD), and skewness (Ske). Upon looking at Table 14, a distinct pattern can be observed in certain spectral and magnitude features across all EMD outputs, including SpBw, ApEn, and ZCR, which is not present in the statistical features. Based on this, the study suggests spectral and magnitude analysis of EEG signals.

Table 14. Summary of spectral, magnitude, and statistical contents for the displayed IMFs and residuals of three different datasets.

| **Dataset** | **EMD Outputs** | **Spectral** | | | **Magnitude** | | | **Statistical** | | |
| --- | --- | --- | --- | --- | --- | --- | --- | --- | --- | --- |
|  |  | **SpCe** | **SpBw** | **SpFt** | **ApEn** | **ZCR** | **CF** | **M** | **SD** | **Ske** |
| **Neurotypical** | IMF1 | 21.7 | 17.4 | 0.21 | 0.97 | 54.5 | 4.01 | 9.4$e^{-6}$ | 5.4$e^{-4}$ | 0.10 |
|  | IMF2 | 10.2 | 8.2 | 0.11 | 0.63 | 20.0 | 3.17 | 2.4$e^{-5}$ | 4.9$e^{-4}$ | 0.03 |
|  | IMF3 | 5.7 | 3.9 | 0.09 | 0.42 | 7.5 | 2.96 | −7.9$e^{-6}$ | 6.3$e^{-4}$ | 0.16 |
|  | IMF4 | 11.8 | 1.6 | 0.31 | 0.16 | 3.6 | 2.76 | −2.1$e^{-5}$ | 4.1$e^{-4}$ | 0.11 |
|  | IMF5 | 2.5 | 0.50 | 0.06 | 0.05 | 1.5 | 2.43 | −3.7$e^{-5}$ | 4.3$e^{-4}$ | 0.05 |
|  | IMF6 | 14.1 | 0.38 | 0.38 | 0.03 | 0.63 | 2.03 | 1.1$e^{-5}$ | 1.5$e^{-4}$ | -0.18 |
|  | IMF7 | 5.3 | 0.13 | 0.14 | 0.01 | 0.25 | 1.61 | 7.3$e^{-6}$ | 6.1$e^{-5}$ | -2.8 |
|  | Residual | 11.0 | 0 | 0.30 | 0 | 0.13 | 1.74 | −1.1$e^{-5}$ | 7.1$e^{-6}$ | -0.05 |
| **Mild AD** | IMF1 | 22.4 | 23.3 | 0.28 | 1.1 | 55.6 | 4.2 | 3.1$e^{-5}$ | 1.3$e^{-3}$ | -0.04 |
|  | IMF2 | 10.6 | 7.5 | 0.13 | 0.63 | 18.9 | 3.3 | $1e^{-5}$ | 1.7$e^{-3}$ | 0.11 |
|  | IMF3 | 9.2 | 4.4 | 0.21 | 0.44 | 7.6 | 2.9 | −9.7$e^{-5}$ | 1.2$e^{-3}$ | 0.01 |
|  | IMF4 | 7.8 | 1.9 | 0.19 | 0.18 | 3.4 | 2.2 | −2.8$e^{-5}$ | 5.4$e^{-4}$ | -0.01 |
|  | IMF5 | 5.5 | 0.5 | 0.14 | 0.05 | 1.1 | 2.6 | $2.6e^{-5}$ | 6.3$e^{-4}$ | 0.37 |
|  | IMF6 | 7.8 | 0.3 | 0.21 | 0.02 | 0.5 | 1.6 | 4.1$e^{-5}$ | 1.5$e^{-4}$ | -0.33 |
|  | IMF7 | 10.7 | 0.1 | 0.29 | 0.01 | 0.3 | 2.2 | 2.5$e^{-5}$ | 9.1$e^{-5}$ | -0.77 |
|  | Residual | 14.0 | 0.1 | 0.38 | 0 | 0.1 | 1.9 | −1.4$e^{-5}$ | 4.1$e^{-5}$ | -0.05 |
| **Moderate AD** | IMF1 | 33.3 | 55.1 | 0.29 | 1.2 | 85 | 4.7 | −4.4$e^{-6}$ | 1.2$e^{-3}$ | 0.006 |
|  | IMF2 | 12.2 | 11.8 | 0.09 | 0.64 | 28.3 | 3.5 | −2.4$e^{-5}$ | 1.4$e^{-3}$ | -0.002 |
|  | IMF3 | 5.2 | 5.3 | 0.02 | 0.54 | 11.4 | 5.0 | −1$e^{-5}$ | 1.1$e^{-3}$ | -0.95 |
|  | IMF4 | 4.7 | 2.1 | 0.09 | 0.23 | 4.1 | 3.4 | 9$e^{-9}$ | 7.9$e^{-4}$ | -0.04 |
|  | IMF5 | 10.7 | 0.8 | 0.29 | 0.07 | 1.6 | 2.3 | −3.9$e^{-5}$ | 5.1$e^{-4}$ | -0.26 |
|  | IMF6 | 7.4 | 0.4 | 0.20 | 0.03 | 0.8 | 1.6 | −2.8$e^{-6}$ | 1.9$e^{-4}$ | -0.02 |
|  | IMF7 | 1.8 | 0.1 | 0.05 | 0.01 | 0.3 | l.4 | 2$e^{-5}$ | 1.6$e^{-4}$ | -0.12 |
|  | Residual | 13.2 | 0.1 | 0.36 | 0 | 0.1 | 2.3 | 3.4$e^{-5}$ | 5.1$e^{-5}$ | 0.46 |

**APPENDIX 2**

Table 15 shows that our study has greater classification accuracy than other studies.

**Table 15.** A comparison of the classification results for AD diagnosis with previous studies.

| **The reference** | **Feature Extraction** | **Classification** | **CV** | **Issue** | **Acc.%** |
| --- | --- | --- | --- | --- | --- |
| **Morabito et al. (2016)** | — | CNN | k-fold | AD vs. MCI | 78 |
|  |  |  |  | NP vs. AD | 85 |
|  |  |  |  | NP vs. MCI | 85 |
|  |  |  |  | NP vs. MCI vs. AD | 82 |
| **Cassani et al. (2017)** | spectral power,  coherence and  amplitude modulation | SVM | k-fold | NP vs. (Mild, Moderate  and Severe AD) | 91.1 |
|  |  |  | LOSO |  | 81.4 |
| **Ieracitano et al. (2020)** | CWT and BiS | MLP, LR, SVM | k-fold | NP vs. MCI | 96.24 |
|  |  |  |  | NP vs. AD | 96.95 |
|  |  |  |  | MCI vs. AD | 90.24 |
|  |  |  |  | NP vs. MCI vs. AD | 89.22 |
| **Trambaiolli et al. (2017)** | Wavelet and  visibility graph | SVM | k-fold | NP vs. (Mild and  Moderate AD) | 91.18 |
|  |  |  | LOSO |  | 85.29 |
| **Simons et al. (2015)** | QSE | LDA | LOSO | NP vs. AD | 77.27 |
| **Fiscon et al.**  **(2018b) and Fiscon**  **et al. (2018a)** | FT, wavelet | J48 | k-fold | NP vs. MCI | 93.3 |
|  |  |  |  | NP vs. AD | 80.6 |
|  |  |  |  | MCI vs. AD | 66.7 |
|  |  |  |  | NP vs. (AD+MCI) | 84.4 |
| **Ruiz-Go´mez et al. (2018a)** | Spectral and  non-linear features | LDA, QDA,  MLP-ANN | LOSO | NP vs. (AD+MCI) | 78.43 |
| **Triggiani et al. (2017)** | eLORETA | ANN | k-fold | NP vs. AD | 76.7 |
| **Houmani et al.**  **(2018)** | Epoch-based  entropy, bump  modeling | SVM | LOSO | NP vs. AD | 91.6 |
| **Maturana-**  **Candelas et al. (2019)** | MSE, rMSSE | QDA | LOSO | NP vs. (Mild, Moderate and Severe AD) | 79.1 |
| **Amezquita-**  **Sanchez et al. (2019)** | MUSIC-EWT with  Fractality dimension | EPNN | k-fold | MCI vs. AD | 90.3 |
| **Kanda et al. (2014)** | Morlet wavelet filter | SVM | k-fold | NP vs. (Mild and  Moderate AD) | 83.95 |
|  |  |  | LOSO |  | 84.56 |
| **Cassani et al. (2014)** | Spectral, Coherence,  and Amplitude modulation | SVM | LOSO | NP vs. (Mild, Moderate  and Severe AD) | 84.7 |
| **Rodrigues et al. (2016)** | DWT | surrogate DT | k-fold | NP vs. MCI vs. AD | 95.45 |
|  |  |  | LOSO |  | 94.88 |
| **Cura et al. (2021)** | EMD, Ensemble  EMD, and DWT | DT, SVM,  KNN, and RF | k-fold | NP vs. AD | 96.5 |
| **Safi and Safi (2021)** | DWT and EMD | SVM, KNN,  and LDA | k-fold | NP vs. Mild AD vs.  Moderate AD | 97.64 |
|  |  |  | LOSO |  | 81.08 |
| **Pirrone et al. (2022)** | FRF and PCA | DT, SVM,  and KNN | k-fold | NP vs. MCI | 97 |
|  |  |  |  | NP vs. AD | 96 |
|  |  |  |  | MCI vs. AD | 83 |
|  |  |  |  | NP vs. (MCI + AD) | 89 |
|  |  |  |  | NP vs. MCI vs. AD | 86 |
| **Alessandrini et al. (2022)** | RPCA and PCA | RNN | k-fold | NP vs. AD | 97.9 |
| **The current**  **work** | **EMD + (ApEn,**  **Energy, LBP, Norm, PPV, and ZCR)** | **LDA, SVM,**  **RF, ANN,**  **RNN, and**  **CNN** | **k-fold** | **NP vs. Mild AD** | **99.8** |
|  |  |  |  | **NP vs. Moderate AD** | **99.9** |
|  |  |  |  | **Mild AD vs. Moderate**  **AD** | **93.6** |
|  |  |  |  | **NP vs. (Mild AD +**  **Moderate AD)** | **99.9** |
|  |  |  |  | **NP vs. Mild AD vs.**  **Moderate AD** | **95.7** |
|  |  |  | **LOSO** | **NP vs. Mild AD** | **93.9** |
|  |  |  |  | **NP vs. Moderate AD** | **94.8** |
|  |  |  |  | **Mild AD vs. Moderate**  **AD** | **88.6** |
|  |  |  |  | **NP vs. (Mild AD +**  **Moderate AD)** | **93.9** |
|  |  |  |  | **NP vs. Mild AD vs.**  **Moderate AD** | **88.2** |
